# Diversity of Natural Self-Derived Ligands Presented by Different HLA Class I Molecules in Transporter Antigen Processing-Deficient Cells

**DOI:** 10.1371/journal.pone.0059118

**Published:** 2013-03-26

**Authors:** Elena Lorente, Susana Infantes, Eilon Barnea, Ilan Beer, Alejandro Barriga, Noel García-Medel, Fátima Lasala, Mercedes Jiménez, Arie Admon, Daniel López

**Affiliations:** 1 Unidad de Procesamiento Antigénico-Inmunología Viral, Centro Nacional de Microbiología, Instituto de Salud Carlos III, Madrid, Spain; 2 Department of Biology, Technion-Israel Institute of Technology, Haifa, Israel; 3 Centro de Biología Molecular Severo Ochoa, CSIC/Universidad Autónoma de Madrid, Madrid, Spain; 4 Unidad de Proteómica, Centro Nacional de Microbiología, Instituto de Salud Carlos III, Madrid, Spain; Centro di Riferimento Oncologico, IRCCS National Cancer Institute, Italy

## Abstract

The transporter associated with antigen processing (TAP) translocates the cytosol-derived proteolytic peptides to the endoplasmic reticulum lumen where they complex with nascent human leukocyte antigen (HLA) class I molecules. Non-functional TAP complexes and viral or tumoral blocking of these transporters leads to reduced HLA class I surface expression and a drastic change in the available peptide repertoire. Using mass spectrometry to analyze complex human leukocyte antigen HLA-bound peptide pools isolated from large numbers of TAP-deficient cells, we identified 334 TAP-independent ligands naturally presented by four different HLA-A, -B, and -C class I molecules with very different TAP dependency from the same cell line. The repertoire of TAP-independent peptides examined favored increased peptide lengths and a lack of strict binding motifs for all four HLA class I molecules studied. The TAP-independent peptidome arose from 182 parental proteins, the majority of which yielded one HLA ligand. In contrast, TAP-independent antigen processing of very few cellular proteins generated multiple HLA ligands. Comparison between TAP-independent peptidome and proteome of several subcellular locations suggests that the secretory vesicle-like organelles could be a relevant source of parental proteins for TAP-independent HLA ligands. Finally, a predominant endoproteolytic peptidase specificity for Arg/Lys or Leu/Phe residues in the P_1_ position of the scissile bond was found for the TAP-independent ligands. These data draw a new and intricate picture of TAP-independent pathways.

## Introduction

Proteolysis, by the proteasome and other cytosolic proteases, of both newly synthesized proteins and the mature cell proteome continuously generates short peptides that are transported into the endoplasmic reticulum (ER) by the transporter associated with antigen processing (TAP) [1]. These peptides are assembled with a nascent HLA class I heavy chain and β2-microglobulin to generate stable HLA/peptide complexes that are exported to the cell membrane and subjected to cytotoxic CD8^+^ T lymphocyte recognition (reviewed in [2]).

Non-functional TAP complexes, which can be produced by mutations in the TAP gene, have been described in both humans [3] and mice [4]. Patients with an HLA class I deficiency have a reduced functional CD8^+^ population but may appear asymptomatic for long periods of time with only a limited susceptibility to chronic respiratory bacterial infections. Thus, their immune systems must be reasonably efficient, and in addition to different unaltered layered defenses, it remains possible that the reduced cytolytic CD8^+^ αβ T subpopulation that is specific for TAP-independent antigens may contribute to immune defenses that protect against severe infections in these individuals.

Although TAP-independent viral epitopes are known (reviewed in [5]–[7]), few studies have analyzed the cellular TAP-independent HLA class I peptide repertoire. TAP-deficient cells have been described as having very limited antigen processing capacity [8], with predominant proteolytic ER signal peptidase (SPase) activity [9]. Therefore, is the TAP-independent HLA peptidome so limited in TAP-deficient cells, as suggested by these studies? The identification of self-derived ligands presented in the same cells by several common HLA antigens with very different TAP dependency is of major interest. Therefore, using a *high-throughput* immunopeptidomics analysis, we analyzed the TAP-independent HLA peptidome isolated from large numbers of TAP-deficient cells and bound to different HLA alleles. In this study, we identified more than three hundred TAP-independent ligands bound to different HLA-A, -B, and -C class I molecules.

## Materials and Methods

### Cell Lines

T2 is a human T cell leukemia/B cell line hybridoma cell line that has a large homozygous deletion within the MHC, including both TAP genes and all of the functional class II genes [10]–[12]. In addition, this cell line expresses low levels of HLA class I molecules on the cell surface [13]. T2 cells transfected with B*2705 have been previously described [14]. Transfected RMA-S tumour of T cells (deficient in TAP that expresses low levels of cell surface MHC class I) expressing HLA-B*2705 have also been previously described [15]. All cell lines were cultured in RPMI 1640 supplemented with 10% fetal bovine serum (FBS) and 5 µM β-mercaptoethanol.

### Synthetic Peptides

Peptides were synthesized in a peptide synthesizer (model 433A; Applied Biosystems, Foster City, CA) and purified by reversed-phase HPLC. The correct molecular mass of each peptide was established with a Reflex IV MALDI-TOF instrument (Brucker-Franzen Analytik, Bremen, Germany), and their correct composition was determined with a Deca XP LCQ mass spectrometer (Thermo Fisher, San Jose, CA).

### Isolation of HLA-bound Peptides

HLA-bound peptides were isolated from 4 x 10^10^ healthy T2-B27 transfected cells or vaccinia (VACV)-WR-infected T2-B27 transfected cells. Cells were lysed at 4°C in 1% CHAPS (Sigma), 20 mM Tris/HCl buffer, and 150 mM NaCl, pH 7.5, in the presence of a protease inhibitor cocktail [16], [17]. First of all, the soluble fraction of cell extracts was treated with immunoaffinity columns without antibody to discard unspecific binding of peptides. Next, HLA-peptide complexes were isolated via affinity chromatography of the soluble fraction of cell extracts with the following mAbs, used sequentially: PA2.1 (anti-HLA-A2) [18], ME1 (anti-HLA-B27) [19], and W6/32 (specific for a monomorphic HLA class I determinant) [20] (Figure S1). HLA-bound peptides were eluted at room temperature with 0.1% aqueous trifluoroacetic acid (TFA), separated from the large subunits and concentrated with a Centricon 3 ultrafiltration device (Amicon, Beverly, MA) exactly as previously described [16], [17].

### Electrospray-Orbitrap Mass Spectrometry Analysis

Peptide mixtures recovered after the ultra-filtration step were concentrated with Micro-Tip reverse-phase columns (C_18_, 200 µl, Harvard Apparatus, Holliston, MA) [16]. Each C_18_ tip was equilibrated with 80% acetonitrile in 0.1% TFA, washed with 0.1% TFA, and then loaded with the peptide mixture. The tip was then washed with an additional volume of 0.1% TFA and the peptides were eluted in 300 µl with 80% acetonitrile in 0.1% TFA. Peptide samples were then concentrated to approximately 18 µl using vacuum centrifugation [16], [17].

HLA class I peptides that had been immunoprecipitated with each HLA-specific mAb were analyzed by µLC-MS/MS using an Orbitrap XL mass spectrometer (Thermo Fisher) fitted with a capillary HPLC (Eksigent, Dublin, CA) [16], [17]. The peptides were resolved on homemade Reprosil C18 capillary columns (75 micron ID) [21] with a 7%–40% acetonitrile gradient for 2 h in the presence of 0.1% formic acid. The seven most intense masses that exhibited single-, double-, and triple-charge states were selected for fragmentation from each full mass spectrum by collision-induced dissociation.

### Database Searches

Raw mass spectrometry data were analyzed using various software tools: Proteome Discoverer 1.0 SP1 (Thermo-Fisher) combining the results of Sequest 3.31 and Bioworks Browser 3.3.1 SP1 (Thermo-Fisher) [22], using the human part of the NCBI database (Feb 2012) including 729,880 proteins. The search was not limited by enzymatic specificity, the peptide tolerance was set to 0.01 Da, and the fragment ion tolerance was set to 0.5 Da [16], [17]. Oxidized methionine was searched as a variable modification. Other search criteria were set such that the search was not limited by any methodological bias (selection of individual protein, use of HLA consensus scoring algorithms, etc.). To exclude peptides that could contaminate the peptide pool, a search using the bovine part of the NCBI database (Feb 2012) including 29,925 proteins was performed. No bovine serum peptides were identified bound to HLA class I molecules.

Identified peptides were selected if the following criteria were met: mass accuracy ≤0.005 Da or <5 ppm; Sequest Xcorr >1.5 for singly, >2.5 for doubly, and >3.5 for triply charged peptides; ΔCn >0.1; Proteome Discovered P score >20; and P(pep) <1×10^−2^ with Bioworks Browser [16], [17]. When the MS/MS spectra fitted more than one peptide, only the highest scoring peptide was selected. The false-positive rate for peptide identification yielded a 2% based on a search of a reversed database, wherein the amino acid sequences of all proteins are reversed. In addition, two synthetic peptides were made, and their MS/MS spectra were used to confirm the assigned sequences (Figures S2 and S3).

### MHC/Peptide Stability Assays

The following synthetic peptides were used as controls in complex stability assays: Flu NP (SRYWAIRTR, HLA-B27-restricted) [23], RSV M_76–84_ (SRSALLAQM, HLA-B27-restricted) [16], and C4CON (QYDDAVYLK, HLA-Cw4-restricted) [24]. RMA-S B*2705 transfectant cells were incubated at 26°C for 16 h in RPMI 1640 medium supplemented with 10% heat-inactivated FBS. This allows the expression of empty MHC class I molecules (without antigenic peptide) at the cellular membrane that are stable at 26°C but not at 37°C. The cells were washed and incubated for 2 h at 26°C with various concentrations of peptide in the same medium without FBS. The cells were maintained at 37°C for an additional 4 h and then collected for flow cytometry. This method allows empty MHC class I molecules to become internalized and can thus discriminate between bound and unbound peptides. MHC expression was measured using 100 µl of hybridoma culture supernatant containing ME1 (anti-HLA-B27) mAb as previously described [25]. Samples were acquired on a FACSCanto flow cytometer (BD Biosciences, San Jose, CA, USA) and analyzed using CellQuest Pro 2.0 software (BD Bioscience). Cells incubated without peptide had peak fluorescence intensities close to background staining with secondary Ab alone. The fluorescence index was calculated at each time point as the ratio of the mean channel fluorescence of the sample to that of the control incubated without peptide. The data are mean values of the three experiments.

## Results

### Physiological Processing Generates Multiple Cellular Ligands Bound to HLA Alleles with Distinct TAP Dependency in the Same Human TAP-deficient Cell Line

To date, approximately 70 human TAP-independent ligands from classical HLA class I molecules are known [7], [9], and are mostly restricted to HLA-A2 and derived by cleavage of signal sequences generated by the SPase complex. Thus, the comparison of TAP-independent peptide pools derived from HLA-A2, HLA-B27, an allele high TAP-dependent [26], and other HLA class molecules, such as HLA-B51 or -Cw1, with no data about their TAP dependency, could be relevant in the study of alternative antigen processing pathways.

In a previous study, HLA-A2, -B27, -B51, and -Cw1-bound peptide pools were isolated from large amounts of either healthy or the vaccinia (VACV)-infected human TAP-deficient cell line (Figure S1) and eleven VACV viral ligands were identified [17]. Moreover, the use of several software tools in these same samples over a human proteome database resolved 111, 77, and 192 fragmentation spectra as peptidic sequences of different human cellular proteins bound to HLA-A2 (Table S1), -B27 (Table S2), and -B51 or -Cw1 (Table S3), respectively. Two different peptide sequences were selected as additional controls for assignment. Both the experimentally detected and the corresponding synthetic peptide MS/MS spectra were identical (Figures S2 and S3). To confirm that HLA-B27 is the MHC class I molecule that presents these ligands, MHC/peptide complex stability assays were performed using TAP-deficient RMA-S cells transfected with the HLA-B27 molecule (Fig. S4).

Collectively, these results indicate that a similar broad range of TAP-independent ligands was endogenously processed and presented by different HLA class I molecules in the same infected cells, despite their differences in TAP dependency [26].

### Structural Features of TAP-independent HLA Ligands

HLA-A2, -B51, and -Cw1 class I molecules usually bind peptides approximately 9–11 residues long (SYFPEITHI database: http://www.syfpeithi.de [27]), whereas HLA-B27 could accommodate peptides up to 13–14 residues in a bulged conformation (SYFPEITHI database, [28]). The analysis of size indicated that approximately 60% of the TAP-independent HLA-A2, -B51, or -Cw1 ligands and 40% of the -B27 ligands are longer than those identified in TAP-sufficient cells (Fig. S5). We next studied the anchor motif requirements of these ligands. The HLA-A2, -B51, and -Cw1 alleles present peptides with partially similar anchor motifs (SYFPEITHI database). The classical position 2 anchor motifs are as follows: for HLA-A2 binding, Leu or Met; for HLA-B51, Pro and Ala; and for HLA-Cw1, Ala and Leu. Aliphatic CΩ residues (SYFPEITHI database) are common between these three HLA alleles. For HLA-B27, the anchor motif consists of Arg or Gln at P2 and basic or aliphatic CΩ residues (SYFPEITHI database, [28]). However, the respective anchor motifs were absent in 60–70% of the TAP-independent HLA ligands (Table 1). Likewise, the amino acid preference at the CΩ position was studied and revealed major discrepancies among the HLA-A2, -B51 and -Cw1 ligands of TAP-sufficient versus TAP-deficient cells, although no differences in HLA-B27 ligands were found (Table 1). These data suggest that the relative contribution of the CΩ pocket to the stabilization of unusual peptides differs among these HLA class I alleles.

**Table 1:** Amino acid preference at anchor motif P2 and CΩ positions in TAP-dependent versus TAP-independent ligands.

Position	Residue	HLA-A2		Residue	HLA-B27		Residue	HLA-B51, Cw1	
		TAP^+a^	TAP^−b^		TAP^+c^	TAP^−d^		TAP^+e^	TAP^−f^
P2	L/M	73	42	R/Q	100	28	L/A/P	81	39
CΩ	L/V/I/A	92	41	R/F/K/L/Y	91	78	V/I/L	83	31

a 424 HLA-A2 ligands from SYFPEITHI database.

b 111 HLA-A2 TAP-independent ligands, see Table S1.

c 571 HLA-B27 ligands [28].

d 77 HLA-B27 TAP-independent ligands, see Table S2.

e 68 HLA-B51 and 9 HLA-Cw1 ligands from SYFPEITHI database.

f 192 HLA-B51 and -Cw1TAP-independent ligands, see Table S3.

g Data are expressed in percentage.

It is well documented that HLA*-*A2 binds signal sequence-derived peptides generated by the cleavage of signal sequences from the parental polypeptide by the signal peptidase (SPase) complex [9]. We found that 11% of peptides that bound to the HLA-A2 molecule were derived from the signal sequence of various proteins (Table 2). In addition, 3% of HLA-B27 and -B51 or -Cw1 ligands are located in the region generated by SPase activity (Table 2). Most importantly, a significant fraction of bound ligands identified from HLA-A2 (17%)-, -B27 (23%)- and -B51 or -Cw1 (24%)-associated repertoires are located at the C-terminal position of their respective proteins. Thus, only one endoproteolytic cleavage was needed to release these particular ligands. In contrast, the remainder of peptides required two endoproteolytic cleavages for their generation. Unexpectedly, a similar fraction of the double cleaved HLA ligands were nested set peptides with an identical core but with N- and/or C-extended residues from the same protein (Table 2 and Tables S1, S2, and S3). Representative nested set peptides are depicted in Figure S6.

**Table 2:** Major features of TAP-independent HLA ligands.

Type of peptide	HLA-A2	HLA-B27	HLA-B51, -Cw1
Signal sequence^a^	11^b^	3	3
C-terminal^c^	17	23	24
N-extended^d^	10	18	11
C-extended^e^	4	8	4
N- and C-extended	1	0	1
Total of extended	15	26	16

a Peptides located into signal sequence of respective protein.

b Data are expressed in percentage of total TAP-independent ligands.

c Peptides located in C-terminal position of respective protein.

d N-extended peptides respect the minimal ligand identified with identical core.

e C-extended peptides respect the minimal ligand identified with identical core.

### TAP-independent Processing Generates Multiple HLA Ligands from the same Cellular Proteins

**Figure 1:**
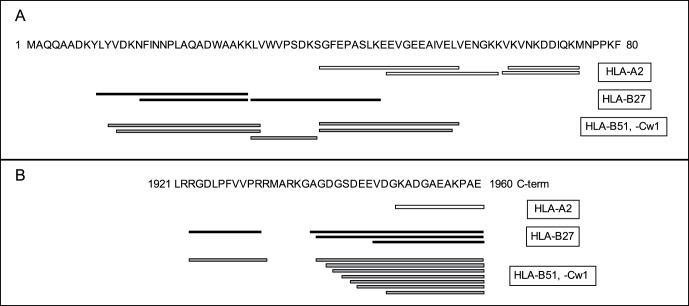
Naturally processed peptides from myosin heavy polypeptide 9 identified by mass spectrometry. Diagram of identified ligands bound to HLA class I molecules in the first 80 (panel A) or last 40 (panel B) residues from myosin heavy chain 9 protein. Ligands specific for HLA-A2 (white boxes), -B27 (black boxes), and –B51 or –Cw1 (gray boxes) are depicted in the lower section of each panel.

In addition to N- and C-extended peptides (Tables S1, S2 and S3, and Figure S6), some proteins contributing different clustered HLA ligands were identified by mass spectrometry (Tables S1, S2, S3, and S4). Figure 1 depicts a representative example. In the first 80 residues of myosin heavy polypeptide 9 protein, different adjacent or superimposed HLA ligands were processed. In addition, other N- and C-terminal extended peptides that were identified as binding to different HLA class I molecules were identified from the same protein (Figure 1, panel B). Thus, these data indicate that extensive processing of N- and C-terminal regions of some proteins occurs via TAP-independent antigen processing pathways. These recurrent endoproteolytic activities were not restricted based on their location within the polypeptide because several HLA ligands were identified that constitute internal regions of some proteins (Figure S7 and Table S4).

In total, one-fifth of the identified proteins were processed, and their ligands were later presented by two different HLA class I molecules. Approximately 6% of the identified proteins generated multiple TAP-independent ligands associated with HLA-A2, -B27, and -B51 or –Cw1 class I molecules (Table 3 and Tables S4 and S5).

**Table 3:** HLA restriction and number of proteins from TAP-independent ligands.

Number of HLA alleles	% of Proteins^a^	% of TAP-independent ligands^a^
One	75	45
Two	19	22
HLA-A2 and –B27	2	2
HLA-A2 and –B51 or –Cw1	11	10
HLA-B27 and –B51 or –Cw1	6	10
Three	6^b^	33

a of the total shown in Tables S1, S2, and S3.

b see Table S5.

Collectively, these data indicate that some proteins could be widely processed by proteases via TAP-independent pathways, yielding multiple peptides that could be presented by different HLA class I molecules. Thus, these data suggest that greater proteolytic processing occurs than previously supposed in TAP-independent antigen processing pathways.

### Cellular Location of Parental Proteins for TAP-independent HLA Ligands

The 334 TAP-independent ligands identified by mass spectrometry arose from 182 parental proteins. Table 4 shows the predicted organelle location of these proteins based on the Gene Ontology database (http://www.geneontology.org) [29]. One-third of the parental proteins were from the ER (8%), Golgi (4%), plasma membrane (14%), and secretory granules (6%), which likely represents their direct processing from transported proteins by resident proteases in the HLA-loading compartments. Strikingly, approximately two-thirds of the parental proteins were from non-secretory subcellular compartments, such as the nucleus (32%), cytoplasm (22%), cytoskeleton (9%), and mitochondria (5%). As such, their respective proteins and/or processed ligands must be transported into the ER lumen by a yet unknown pathway to yield TAP-independent antigen presentation.

**Table 4:** Distribution of proteins by cell location.

Location	TAP^−^peptidome	Lysosomes^a^	Secretory vesicles^b^
Cytoplasm	22^c^	16	23
Cytoskeleton	9	0	8
Endoplasmic reticulum	8	2	4
Extracellular	0	0	5
Golgi	4	1	2
Mitochondria	5	2	6
Nucleus	32	5	22
Plasma membrane	14	2	24
Secretory granule^d^	6	72	8

a from human T cells [30], [31].

b from human neutrophils [32].

c data are expressed in percentage of proteins listed by cell location based on gene ontology analysis (http://www.geneontology.org) [29].

d Secretory granule are represented by melanosomes, lysosomes, platelet granules, endosomes, synaptosomes, exosomes or cytolytic granules as defined in references [30], [31].

### The Secretory Vesicle-like Organelle could be an Important Source of Parental Proteins for HLA TAP-independent Ligands

Various TAP-independent ligands from several lysosomal proteins (e.g., lysosomal multispanning membrane protein 5, Figure S6) were identified. Thus, an attractive hypothesis arose that pointed to protein digestion within this degradative organelle as the source of most of the parental proteins yielding the TAP-independent ligands identified by mass spectrometry. Thus, the distribution of both the source proteins of the TAP-independent peptidome and those of the lysosomes previously published [30], [31] was compared. Table 4 shows a very different pattern of organelle location of constitutive proteins between this subcellular organelle and the TAP-independent peptidome. Thus, the lysosomes are not likely to be the primary source of peptides in the TAP-independent peptidome. As some lysosomal proteins are expressed in other secretory organelles, and because diverse vesicular organelles harbor different organelle-specific constitutive proteins, the composition of several organelles was analyzed. Only secretory vesicles from human neutrophils [32], similar to those identified in several immune cells including T and B cells) [33], [34], have shown a similar predominance of cytoplasm, nucleus and plasma membrane proteins in the ratios observed for the TAP-independent peptidome (Table 4). Further, 51% of the parental proteins identified as part of the TAP-independent peptidome (Table S6) were previously found in the proteome of this secretory vesicle [32]. Thus, secretory-like vesicles could be a relevant source of the parental proteins observed during TAP-independent antigen processing.

### Cleavage Specificity of Peptidases Over TAP-independent Ligands

**Figure 2:**
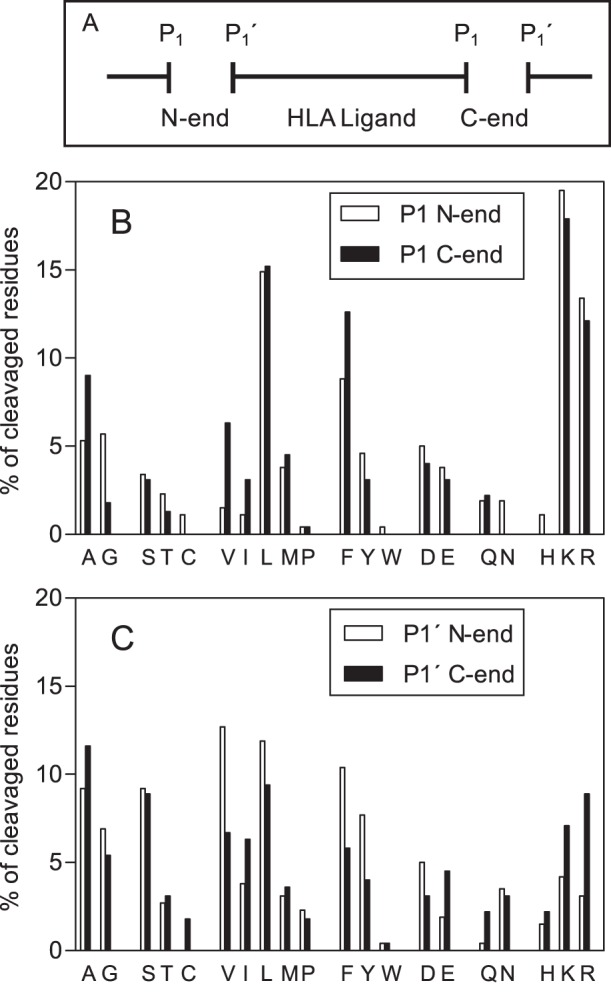
Analysis of N- and C-end cleavage specificity in HLA class I ligands. A diagram of residues involved in the generation of naturally processed HLA class I ligands by peptidase cleavages is shown (panel A). Distribution of P_1_ (panel B) and P′**_1_** (panel C) amino acid residues of the scissile bonds created by peptidase cleavage.

**Figure 3:**
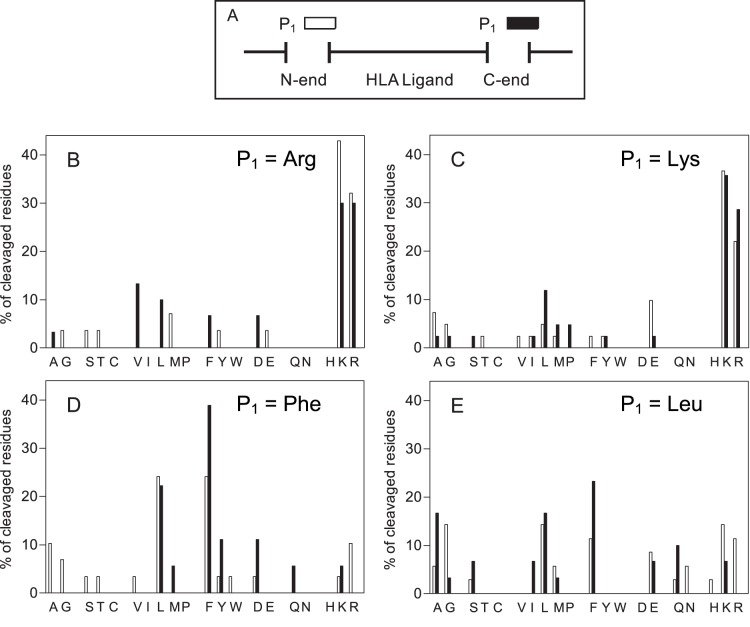
Analysis of the correspondence of P_1_ N- and C-end cleavage specificity in HLA class I ligands. Panel A: A diagram of the residues involved in the generation of naturally processed HLA class I ligands by peptidase cleavage is shown. P_1_ residue is indicated by white boxes (N-end) or black boxes (C-end). Panels B-E: A specific amino acid residue is indicated at the top right corner of each panel and the corresponding opposite residue identified in P_1_ N-end (white boxes) or C-end (black boxes) is represented. For example, panel B indicates in white bars the residue located in the P_1_ N-end position when Arg was identified in P_1_ C-end, and in black bars the residue located in the P_1_ C-end position when Arg was identified in the P_1_ N-end position.

Next, to study the specificity of peptidases involved in the generation of ligands in TAP-defective cells, an analysis was carried out that used mass spectrometry to detect residues on both sides of the hydrolyzed bonds of HLA ligands. When several nested peptides were found, amino and/or carboxyl peptidase activities could be assumed. Accordingly, only the higher possible HLA ligand was examined. Under this hypothesis, 264 TAP-independent ligands (the 79% of peptidome) were analyzed. Figure 2 shows the distribution of amino acids found in the immediate flanking positions of scissile bonds (P_1_ or P′_1_ residues). The relative abundance of most amino acids was similar when P′_1_, but not P_1_, residues of HLA ligands were analyzed (panel B versus C). This was also true when the analysis was performed on each individual HLA class I molecule studied (data not shown). Additionally, no correlation was found when similar analyses of P_2_, P_3_, P′_2_, and P′_3_ positions were performed (data not shown). In summary, these results indicate that proteases with specificity to some residues in P_1_, but not other positions, make the endoproteolytic cleavages to generate TAP-independent ligands. Only four amino acids (Arg, Leu, Lys, and Phe) are increased in the P_1_ positions of scissile bonds, which accounted for up to 10% of the total cleavages detected (Fig. 2, panel B). These four major P_1_ residues could be processed by different proteases or may represent the specificities of one or a few proteases that generate both P_1_ cleavage positions. To resolve these questions, an analysis of the correlation between these four specific amino acid residues and the opposite P_1_ N- or C-end residues of HLA ligands was carried out. When Arg was the P_1_ C-end of an HLA ligand identified by mass spectrometry, only two amino acid residues (Arg and Lys) were predominantly located in the corresponding P_1_ N-end position (Fig. 3B, white boxes). This was true also for the reverse situation; when Arg was the P_1_ N-end position, the amino acid residues predominantly located in the P_1_ C-end position were also Arg and Lys (Fig. 3B, black boxes). Further, an identical correspondence in the analysis of Lys residue was found with both the P_1_ N- and C-end positions (Fig. 3C). Similar analyses with Phe P_1_ cleavages have shown that only Phe and Leu residues were mainly located in the equivalent P_1_ N- or C-end positions of scissile bonds (Fig. 3D). A minor correlation with the Leu cleavage analysis was found, although Phe and Leu remained as the most abundantly detected residues (Fig. 3E). As several endoproteolytic peptidases have specificity for Arg/Lys or Leu/Phe in the P_1_ position of the scissile bond (MEROPS database: http://merops.sanger.ac.uk [35]), the enzymatic activity of only two of these types of peptidases could explain more than half of the identified cleavages derived from the detected TAP-independent ligands (Table S7).

### Low Hydrophobicity in TAP-independent Virus Ligands

In cells infected with the Epstein-Barr virus (EBV), only peptides with high hydrophobicity from the BRLF1 and LMP2 proteins were processed by TAP-independent pathways [36], [37]. In contrast, hydrophobicity is not a necessary condition for the TAP-independent presentation of vaccinia virus ligands [17]. Because the total number of viral ligands in both studies was low (eight and eleven ligands from EBV or vaccinia virus, respectively), the study of the hydrophobicity of more abundant cellular TAP-independent ligands could be important. The grand average of hydropathicity (GRAVY) (ProtParam tool, ExPASy Proteomics Server, http://www.expasy.ch) mean of approximately 1,000 HLA-A2, -B27, -B51, and -Cw1 TAP-dependent ligands previously described (Table 1) was only 0.0±1.0, and significant differences between the different HLA alleles studied were not found (data not shown), indicating that no hydrophobicity of these HLA ligands exists. A very similar GRAVY measurement (−0.3±0.8) was found for the 337 TAP-independent ligands described in this report. These results show that hydrophobicity is not a necessary condition for the overall TAP-independent presentation of cellular ligands.

## Discussion

Identification of self-derived HLA ligands by mass spectrometry analysis contributes to a better understanding of the mechanisms of antigen presentation that are associated with the cellular immune response. While several hundred peptides bound to specific MHC class I alleles are identified in any given immunoproteomics analysis from TAP-sufficient cells, only some tens have been described for TAP-independent HLA ligands from the still very limited number of immunoproteomics studies from TAP-deficient cells. In the first study, 22 and 27 cellular peptides bound to the murine H-2K^d^ class I molecule were immunoprecipitated from human and mouse TAP-deficient cells respectively [8]. Later, 50 HLA-A2 or -B51 ligands were presented TAP-independently in a human TAP-deficient cell line [9]. Currently, using a sequential immunoprecipitation of several HLA class I molecules, we identified several hundred TAP-independent ligands that were processed and presented by the four class I molecules expressed in the same cell population**.** Thus, the HLA peptidome generated by TAP-independent antigen processing pathways is more diverse than previously assumed. In agreement with previous studies [8], [9], the major features of the peptide repertoire bound to classical MHC class I from TAP-deficient cells revealed increased peptide lengths and a lack of strict binding motifs in all HLA class I molecules studied in the current report. The cytosol is a very degradative compartment, having multiple endoproteases and exopeptidases [1]. In contrast, both the vesicular compartments and the secretory pathway (source of TAP-independent ligands) are clearly less degradative [38]. Thus, the absence of high affinity ligands, generated by the TAP-dependent antigen processing, allows the interaction between the high amounts of empty HLA class I molecules and the long TAP-independent ligands with low affinity from TAP-deficient cells [8], [39]. In addition, in a previous study using the same HLA peptidome, several vaccinia low affinity ligands were identified that did not conform to the normal anchor motifs used by HLA-A2, -B27 and –Cw1 class I molecules [17]. Nonetheless, the identified HLA-A2 low-affinity ligand generated long-term CTL memory responses against vaccinia virus, even in an HLA-A2 transgenic TAP**^+^** mouse model [17]. These data and other studies [40], [41] indicate that low-affinity ligands, such as the self-derived peptides identified in the current report, have functional relevance.

Immunoproteomics analysis of the MHC class II peptidome usually shows ligands represented by several length variants of the same core, thereby forming sets of nested peptides varying by several residues at the N- or C-terminal ends (summarized in SYFPEITHI database). Further, in the analysis of the TAP-independent peptide repertoire that is associated with non-classical MHC class I molecules, HLA-E- [39] and H-2Qa-1^b^-bound peptides [42] were identified as length variants of the same central core. In the current report, multiple sets of up to ten nested peptides were endogenously presented by different HLA class I molecules (Table S4), suggesting that amino acid trimming at both the N- and C- terminus might be a general attribute of TAP-independent peptides. The similarities between the classical and non-classical peptide repertoires of HLA class II and TAP-independent HLA class I suggest at least a partial contribution of TAP-independent peptide loading in the post invariant chain-degrading compartments as indicated the existence of uncharacterized TAP-independent peptide-HLA-B27 complexes generated by a chloroquine-sensitive pathway [43].

Unlike previous studies [8], [9], the higher number of ligands identified in the current report allows the identification of extensive TAP-independent antigen processing mechanisms, yielding multiple HLA ligands in a reduced subgroup representing a small number of cellular proteins. Previously, autophagic processes present at low levels in different cell lines have been suggested to explain TAP-independent antigen processing [44]. In addition, as peptides derived from cytosolic proteins are found bound to class II MHC molecules [45]–[47], the mechanisms involved in peptides and/or protein transport from the cytosol to secretory pathways could be similar for both TAP-independent class I and class II MHC ligands. This possibility is supported by our identification of nested sets of TAP-independent peptides that are very similar to those previously reported to be associated with HLA class II molecules. The similar composition of the secretory vesicle proteome that includes both HLA class I and class II molecules [32], as well as the nested set TAP-independent ligands identified in the current report, suggest that the antigen processing of proteins from these or similar organelles could be a relevant source of the TAP-independent peptidome. This vision of MHC class I molecules, from cell surface protein recycling, to entering into classical MHC class II compartments and later being transported back to the plasma membrane associated with endocytic/secretory vesicle peptides, might be the case for the peptides identified in the current report and is supported by two previous studies. At first, a measles virus F protein epitope was previously presented by class I molecules in TAP-independent, acidic-sensitive manner [48]. Second, uncharacterized TAP-independent peptide-HLA-B27 complexes were generated by a proteasome-independent, but chloroquine-sensitive, pathway [43]. Thus, this endocytic/secretory pathway may exist under normal conditions where it may contribute to a minor fraction of presented ligands. However, when the highly predominant TAP-dependent ligands are absent, as in TAP-deficient cells, this process may predominate.

Individual HLA class I alleles have shown different TAP dependencies. The prevalent HLA-A2 allele is considered to be the least TAP-dependent [49]. In contrast, other MHC class I molecules, including HLA-A3, -A24, and -B27, have been described as mainly TAP-dependent [26]. In the present report, a similarly broad TAP-independent peptidome was identified by MS for these HLA alleles with differing TAP requirements. Thus, quantitative rather than qualitative differences (probably associated with the high efficiency rate of the SPase, accounting for the majority of signal sequence cleavages [50]), are responsible for the diverse overall expression of various MHC class I molecules in TAP-deficient cells [43].

**Figure 4:**
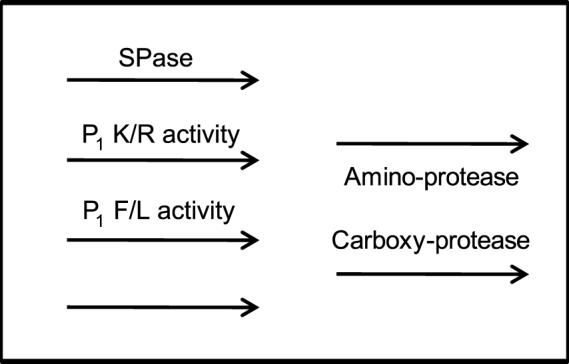
A model of the diversity of proteases and parallel processing pathways involved in TAP-independent self-derived antigen presentation. The model shows the components involved in each of the proposed pathways, with the relative order of the different steps. Involvement of SPase is deduced according to the SwissProt (http://web.expasy.org) and Signal P 4.1 (http://www.cbs.dtu.dk/services/SignalP) predictions. Involvement of P_1_ K/R or F/L activities is deduced from Figures 2 and 3. The lower left arrow is deduced from unassigned ligands, and the amino and/or carboxyl peptidase activities could be assumed by analogy from previous studies.

The global picture emerging from the current report is consistent with the model depicted in Figure 4. Some ligands, mostly HLA-A2-restricted, were processed by the SPase, in accordance with previous studies [9], [43], [51], [52]. Endoproteolytic peptidases, exhibiting specificity for Arg/Lys or Phe/Leu in the P_1_ position of the scissile bond, play an important role in the generation of many ligands associated with the four HLA class I alleles studied herein. However, some TAP-independent ligands must be produced by other, yet uncharacterized protease activities. Finally, a fraction of the longest variants from the same core protein could be folded within the HLA molecules, thereby protecting them from the trimming activity. The remaining molecules might be accessed by amino and/or carboxypeptidases and trimmed to shorter peptides. Several rounds of trimming and HLA/peptide stabilization of fractions of these peptides could form the sets of nested peptides identified by mass spectrometry. Recurrent and sequential amino-terminal trimming and MHC protection have been demonstrated for a nested set of abundant and equally antigenic murine HLA class I epitopes from TAP-sufficient cells that ranged between 9–15 residues [53], [54]. Whereas the role of the ER-resident peptidase ERAP in the N-terminal end trimming of different MHC class I ligands was previously well defined [55]–[57], to date, very few studies have implied a role for carboxypeptidases in antigen processing in the vesicular pathway. Indirect evidence has been reported in two cases. First, the proteolytic action of furin in the secretory pathway is required to generate an antigenic viral epitope [58]. After cleavage by furin, several C-terminal residues must be trimmed from the precursor peptide to generate the optimal epitope, suggesting that carboxypeptidases are involved. Second, several signal sequence-derived peptides generated by SPase complexes have C-terminal-extended residues when compared to the optimal HLA-bound epitope [8], [9], [42], indicating that carboxypeptidases may be involved in antigen processing of these ligands. A direct role for the carboxypeptidase ACE was described for the processing of peptides for MHC class I [59]. Recently, undefined carboxypeptidases were involved in the antigen processing of a vaccinia-derived TAP-independent epitope [60]. Finally, the present report indirectly implicates carboxyproteases in the generation of nested sets of peptides bound to several HLA class I alleles.

In summary, different and complex processing pathways are required to generate the HLA class I peptide repertoire in TAP-deficient cells.

## Supporting Information

Figure S1 **Diagram of sequential immunoprecipitation.**
(PDF)

Figure S2 **Identification of the SF_328–336_ ligand in cell extracts by mass spectrometry.**
(PDF)

Figure S3 **Identification of the LMMP5_249–262_ ligand in cell extracts by mass spectrometry.**
(PDF)

Figure S4 HLA-B*2705 stabilization assay with synthetic ligands.
(PDF)

Figure S5 **Length distribution of naturally processed peptides presented by HLA class I molecules in a TAP-deficient T2 cell line versus TAP-dependent ligands.**
(PDF)

Figure S6 **Representative nested set peptides of ligands identified by mass spectrometry.**
(PDF)

Figure S7 **Naturally processed peptides from MRCL2, β-actin, and glyceraldehyde 3-P dehydrogenase proteins identified by mass spectrometry.**
(PDF)

Table S1 **Summary of HLA-A2 ligands identified by mass spectrometry analysis.**
(PDF)

Table S2 **Summary of HLA-B27 ligands identified by mass spectrometry analysis.**
(PDF)

Table S3 **Summary of HLA-B51, or -Cw1 ligands identified by mass spectrometry analysis.**
(PDF)

Table S4 **Summary of HLA ligands clustered by protein origin.**
(PDF)

Table S5 **Subcellular location of proteins with TAP-independent ligands presented by HLA-A2, -B27, and -B51 or –Cw1.**
(PDF)

Table S6 **Summary of HLA ligands identified and coverage protein from individual protein.**
(PDF)

Table S7 **Summary of predominant cleavaged residues by peptidases in TAP-independent ligands.**
(PDF)
