# Supplementary figures and images for: Diversity of Natural Self-Derived Ligands Presented by Different HLA Class I Molecules in Transporter Antigen Processing-Deficient Cells
Source: PLoS One. 2013 Mar 26;8(3):e59118. doi: 10.1371/journal.pone.0059118 (PMC3608615; doi:10.1371/journal.pone.0059118)

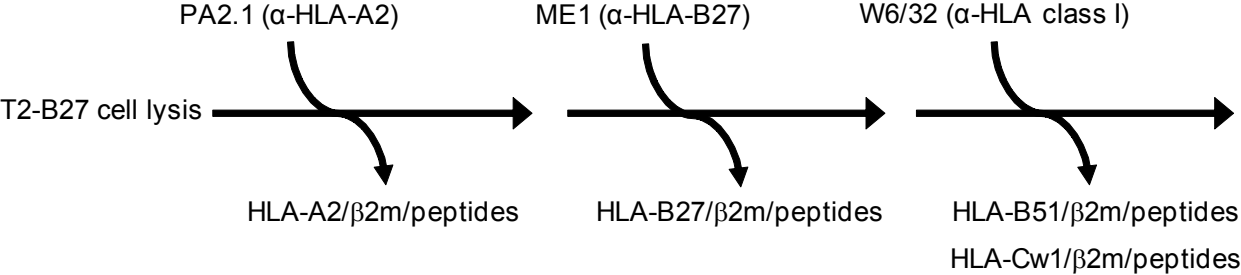

Supplement: Figure S1 — Diagram of sequential immunoprecipitation. (PDF) [file pone.0059118.s001.pdf]

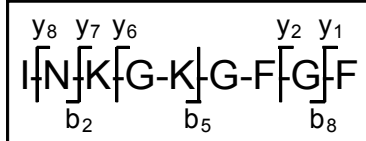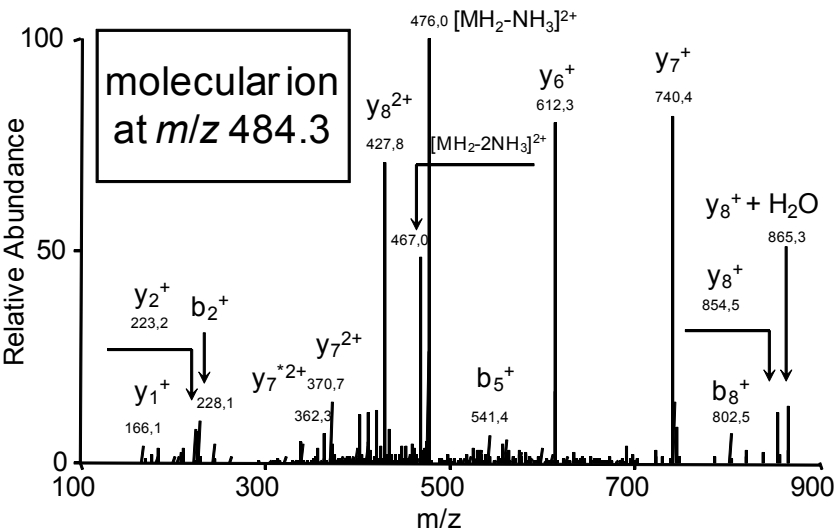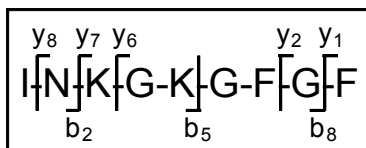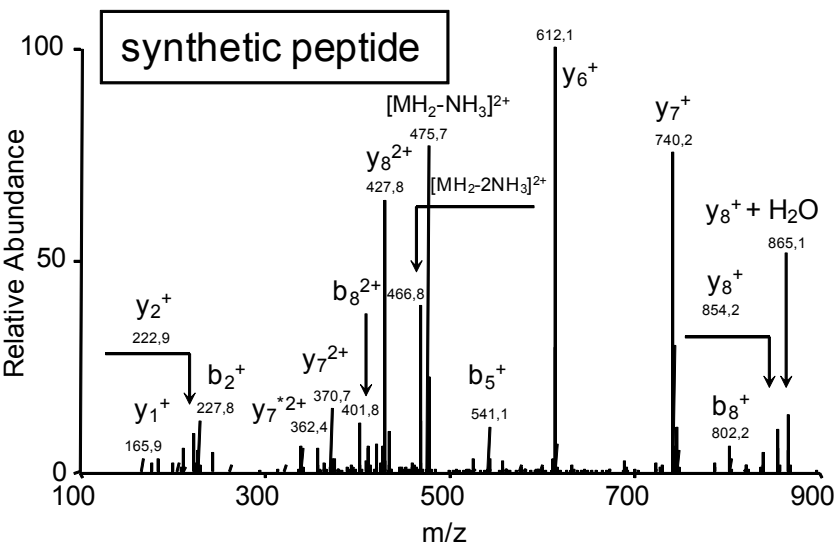

Supplement: Figure S2 — Identification of the SF328–336 ligand in cell extracts by mass spectrometry. (PDF) [file pone.0059118.s002.pdf]

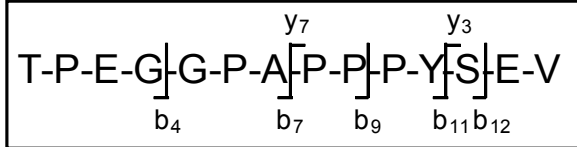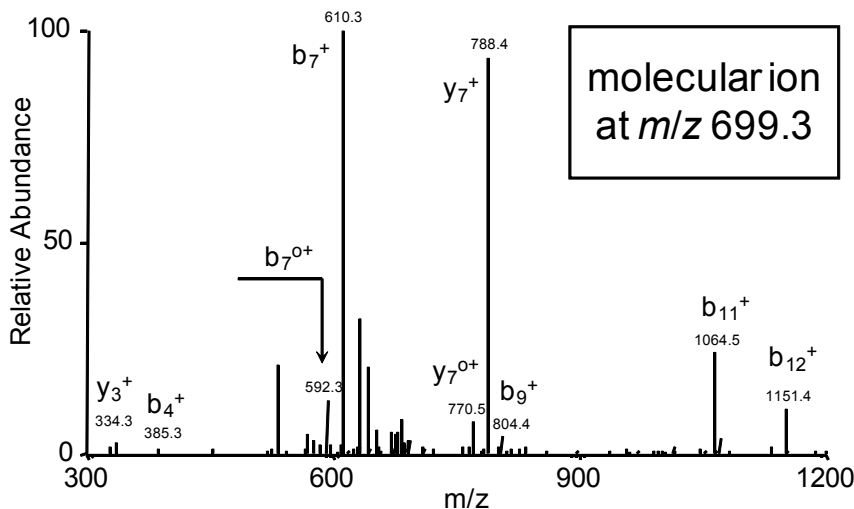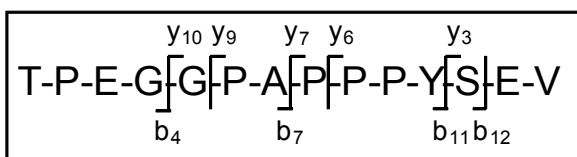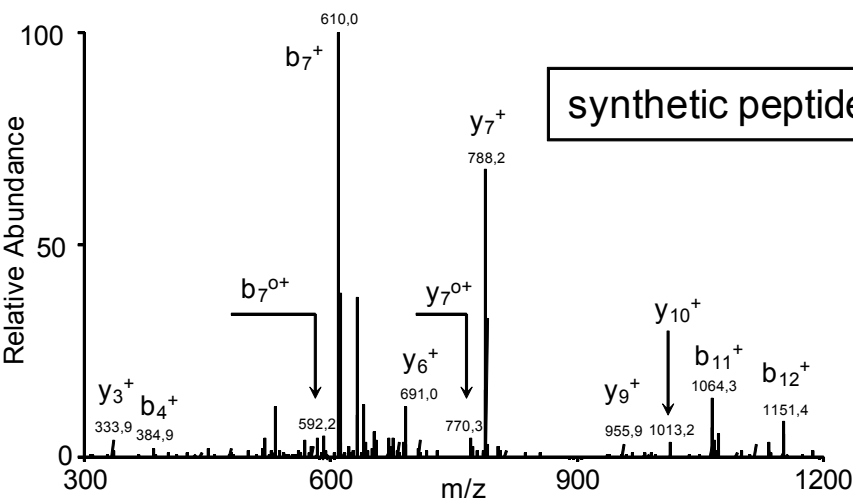

Supplement: Figure S3 — Identification of the LMMP5249–262 ligand in cell extracts by mass spectrometry. (PDF) [file pone.0059118.s003.pdf]

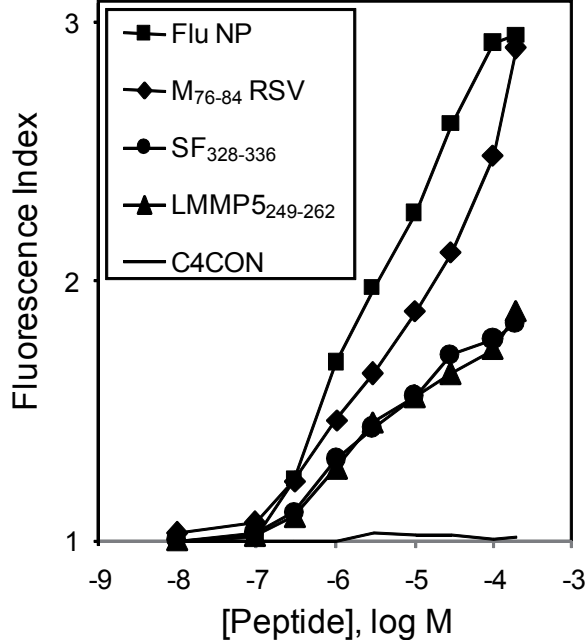

Lorente et al. Figure S4

Supplement: Figure S4 — HLA-B*2705 stabilization assay with synthetic ligands. (PDF) [file pone.0059118.s004.pdf]

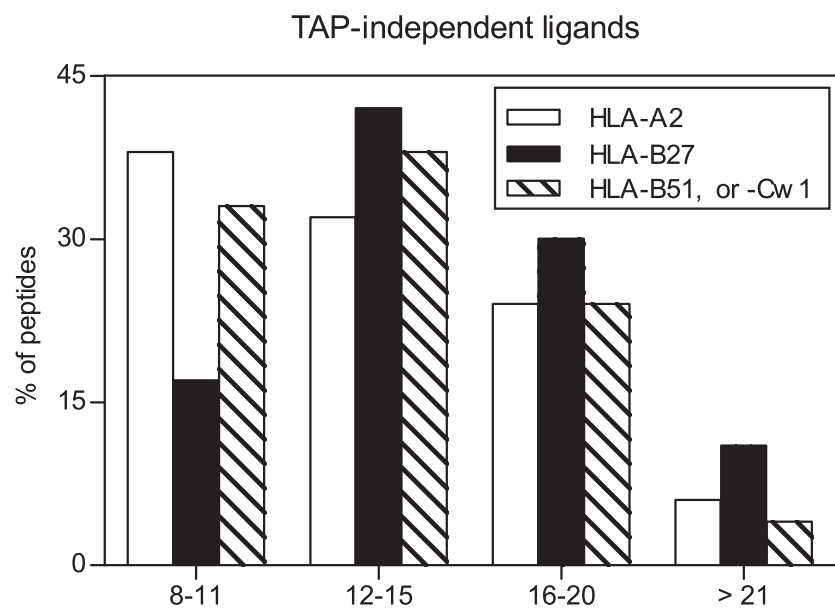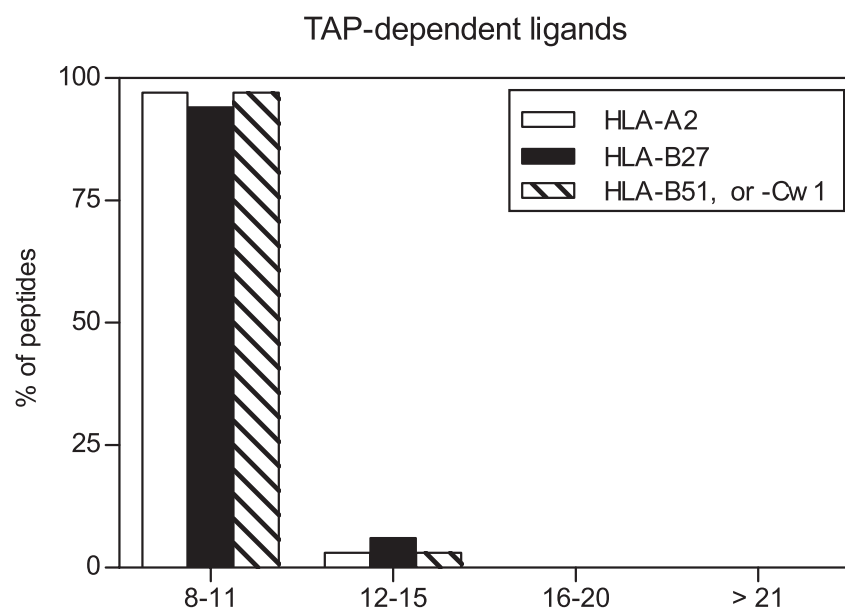

Lorente et al. Figure S5

Supplement: Figure S5 — Length distribution of naturally processed peptides presented by HLA class I molecules in a TAP-deficient T2 cell line versus TAP-dependent ligands. (PDF) [file pone.0059118.s005.pdf]

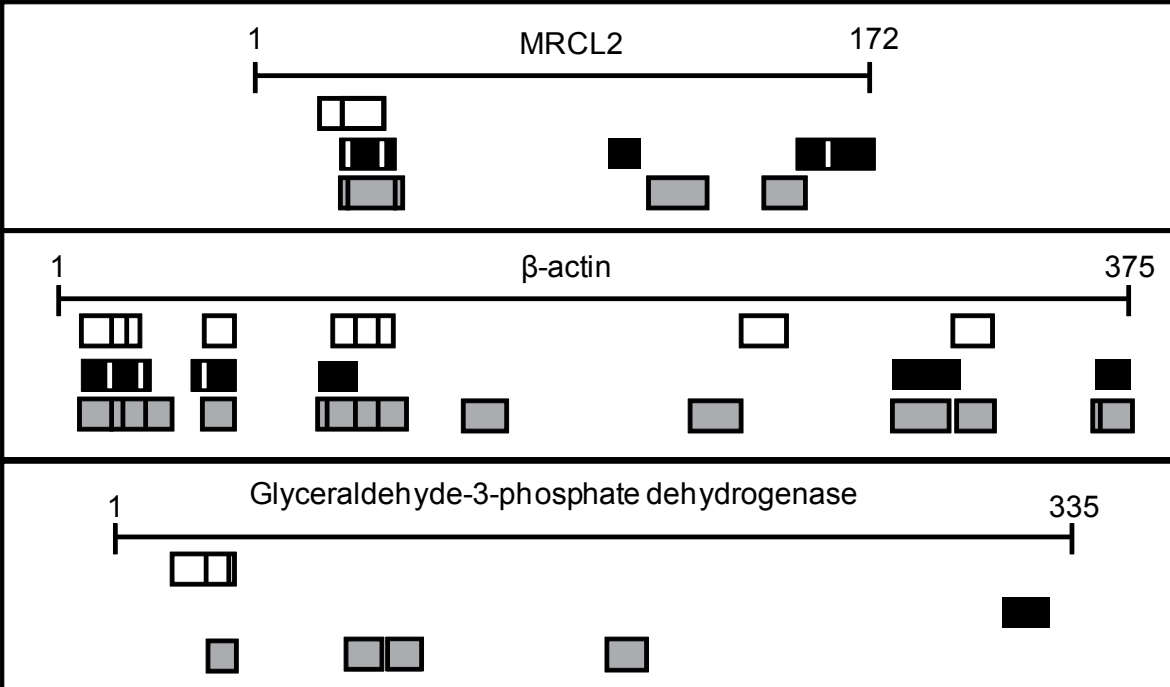

Lorente et al. Figure S7

Supplement: Figure S7 — Naturally processed peptides from MRCL2, β-actin, and glyceraldehyde 3-P dehydrogenase proteins identified by mass spectrometry. (PDF) [file pone.0059118.s007.pdf]
