# Supplementary material for: Diversity of Natural Self-Derived Ligands Presented by Different HLA Class I Molecules in Transporter Antigen Processing-Deficient Cells
Source: PLoS One. 2013 Mar 26;8(3):e59118. doi: 10.1371/journal.pone.0059118 (PMC3608615; doi:10.1371/journal.pone.0059118)
Supplement: Figure S6 — Representative nested set peptides of ligands identified by mass spectrometry. (PDF) [file pone.0059118.s006.pdf]

Lysosomal multispanning membrane protein 5

PSYEEALSLPSKTPEG GPAPPPYSEV
EEALSLPSKTPEG GPAPPPYSEV
EALSLPSKTPEG GPAPPPYSEV
LSLPSKTPEG GPAPPPYSEV
SLPSKTPEG GPAPPPYSEV
LPSKTPEG GPAPPPYSEV
PSKTPEG GPAPPPYSEV
SKTPEG GPAPPPYSEV
KTPEG GPAPPPYSEV
TPEG GPAPPPYSEV
GGPAPPPYSEV
GPAPPPYSEV

HLA-A2

F IAVGYVDDTQ F
IAVGYVDDTQ
IAVGYVDDTQ F
IAVGYVDDTQ FVRF
IAVGYVDDTQ FVRFD
VGYVDDTQFVRFDSD
VGYVDDTQF
VDDTQFVRFDSD

MRCL2

ATSNVFAM FDQSQIQEFK
AMFDQSQIQEFK
AMFDQSQIQEFK EAF
AMFDQSQIQEFK EAFNM
FDQSQIQEFK
FDQSQIQEFK EAFNM

Ig kappa chain precursor

DIVLTQSPASL
DIVLTQSPASL A
DIVLTQSPASL AVSLGQ
DIVLTQSPASL AVSLGQR
DIVLTQSPASL AVSLGQRA

Lorente et al. Figure S6
