# Supplementary material for: Diversity of Natural Self-Derived Ligands Presented by Different HLA Class I Molecules in Transporter Antigen Processing-Deficient Cells
Source: PLoS One. 2013 Mar 26;8(3):e59118. doi: 10.1371/journal.pone.0059118 (PMC3608615; doi:10.1371/journal.pone.0059118)
Supplement: Table S1 — Summary of HLA-A2 ligands identified by mass spectrometry analysis. (PDF) [file pone.0059118.s008.pdf]

Supplemental Table 1. Summary of HLA-A2 ligands identified by mass spectrometry analysis

HLA-A2 ligand sequence	Experimental mass [a]	ΔMass [b]	ΔMass [c]	z	P (pep) [d]	Xcorr [e]	ΔCn [e]	Gi accession	Protein	Position	Length of protein	Sample [f]
SQIDDLYSTIKV	1381.721	-0.005	-3.66	2	5.21E-05	3.1	0.2	40807491	ACSL1	687-698	698	N/I
LLFSAFSRA	1011.562	-0.001	-1.14	2	3.29E-04	2.5	0.2	47125451	ALG10 Protein	22-30	473	N
YGAEALERMFL	1299.640	-0.001	-0.47	2	6.34E-07	3.0	0.3	4504345	Alpha 2 globin	25-35	142	N/I
FELFPSLSHNLLVD	1630.848	-0.001	-0.33	2	6.13E-05	2.9	0.3	17367145	Aspartyl aminopeptidase	462-475	475	N
ALPENPPAI	921.504	-0.003	-3.40	1	4.35E-03	1.8	0.2	5453559	ATP synthase	44-52	161	N
AGFAGDDAPR	976.448	-0.005	-4.88	2	7.02E-07	2.9	0.3	4501885	Beta Actin	19-28	375	N
SYELPDGQVITIGNER	1790.892	-0.004	-2.37	2	3.29E-08	4.5	0.4	4501885	Beta Actin	239-254	375	N/I
MQKEITALAPSTMK	1548.812	0.000	-0.25	2	9.77E-09	4.3	0.3	4501885	Beta Actin	313-326	375	N
VAPEEHPVLLTEAPLNPK	1954.064	0.000	-0.17	2	2.56E-06	3.8	0.4	4501885	Beta Actin	96-113	375	N/I
LVVDNGSGMCKAGFAGDD	1755.768	0.001	0.71	2	1.65E-11	3.8	0.6	4501885	Beta Actin	8-25	375	N
LTEAPLNPKANR	1323.738	0.003	2.55	2	3.88E-05	3.1	0.3	4501885	Beta Actin	105-116	375	N
SGPFGQIFRPDNFVF	1727.854	0.002	0.90	2	7.79E-07	2.8	0.2	2119276	Beta tubulin	71-85	437	N
WQVKSGTIFDNF	1441.711	-0.002	-1.40	2	1.45E-06	2.7	0.3	4757900	Calreticulin precursor	319-330	417	N
FLLSDRSGMTTGSTLPVEGGF	2172.064	0.000	-0.16	2	4.52E-09	4.3	0.4	33150790	Carbonyl reductase II	221-241	244	N
FLYQQQGRLDKLTVT	1809.986	-0.005	-2.57	2	2.55E-05	2.6	0.3	68448544	CD74a	70-84	296	N
FLYQQQGRLDKLTV	1708.938	-0.003	-1.62	2	6.51E-06	3.4	0.4	68448544	CD74a	70-83	296	N
FMDEEEEDEIRV	1540.647	0.001	0.42	2	9.11E-08	3.9	0.5	13786127	Cdc42	345-356	356	N
DVKFGADARALMLQGVDL	1919.006	-0.004	-1.99	2	2.48E-07	3.8	0.3	31542947	Chaperonin	29-46	573	N/I
FGADARALMLQGVDL	1576.815	-0.001	-0.65	2	1.37E-05	2.9	0.3	31542947	Chaperonin	32-46	573	N
FLPTPEELGLLGPPRPQVLA	2144.211	-0.002	-1.06	2	8.04E-13	4.4	0.4	4759328	Class I cytokine receptor	617-636	636	N/I
FLPTPEELGLLGPPRPQVL	2073.174	0.000	0.08	2	1.60E-11	5.1	0.5	4759328	Class I cytokine receptor	617-635	636	N
SLWGQPAEA	958.463	-0.002	-1.87	1	8.88E-04	1.7	0.2	156230677	Collagen, type IV, alpha 5	18-26	1691	N/I
FDIAVDGEPLGRVSFEL	1863.949	-0.003	-1.81	2	2.22E-08	5.0	0.5	1633054	Cyclophilin A	7-23	164	N/I
VDGEPLGRVSFEL	1417.732	-0.003	-2.28	2	2.98E-03	2.8	0.2	1633054	Cyclophilin A	11-23	164	N
IAVDGEPLGRVSFEL	1601.853	-0.001	-0.90	2	8.04E-05	3.4	0.3	1633054	Cyclophilin A	9-23	164	N
FDIAVDGEPLGRVSF	1621.822	0.001	0.36	2	3.52E-10	3.7	0.6	1633054	Cyclophilin A	7-21	164	N
LHLGYLPNQLFRTF	1718.938	-0.001	-0.70	2	6.35E-11	3.9	0.3	4826686	DEAD box polypeptide 1	727-740	740	N/I
TLDEKIEKV	1074.604	0.000	0.17	2	9.63E-04	2.9	0.1	119596071	DEAD box polypeptide 27	85-93	440	N
SYLENPAFMLLDLK	1653.856	-0.002	-1.24	2	3.29E-05	3.5	0.4	736675	Dihydrolipoyl transacylase	464-477	477	I
VLVEPDAGAGV	1026.547	-0.001	-0.98	1	1.56E-03	1.6	0.1	62530384	Dodecenoyl-CoA isomerase	47-57	302	N
FLWNGSGPQGL	1175.584	0.003	2.31	1	5.61E-05	2.0	0.3	62512143	ETS translocation variant 3	495-505	512	N
ALPGDNVGFNV	1102.553	0.000	-0.14	2	1.40E-04	3.0	0.5	4503471	Eukaryotic translation elongation factor 1 alpha 1	302-312	462	N/I
SGKKLEDGPKFLK	1446.831	0.000	-0.07	2	6.14E-05	3.5	0.4	4503471	Eukaryotic translation elongation factor 1 alpha 1	383-395	462	N/I
GFGDLKSPAGLQVLNDY	1793.907	-0.005	-2.69	2	3.14E-08	4.7	0.6	4503477	Eukaryotic translation elongation factor 1 beta 2	2-18	225	N
EGIPALDNFLDKL	1444.768	-0.003	-2.29	2	1.62E-03	3.7	0.3	4503483	Eukaryotic translation elongation factor 2	846-858	858	N
YVQDYEDFM	1209.477	-0.002	-1.56	1	2.46E-04	2.4	0.3	83281438	Eukaryotic translation initiation factor 3	250-258	258	N
YLDLFGDPSV	1125.546	-0.004	-3.58	1	1.06E-03	1.8	0.3	61680786	Farnesyl Diphosphate Synthase	266-275	374	N
ALSDLEITLEGGK	1345.721	0.000	-0.22	2	8.45E-05	3.1	0.2	29789006	Fermitin family homolog 2	349-361	680	N
VGLGLGYLELPQINYK	1776.989	0.000	0.22	2	1.08E-07	4.0	0.4	4503743	Flightless I homolog	731-746	1269	N
GIVEFSGKPAAR	1231.679	-0.002	-2.00	2	2.26E-04	2.5	0.4	348239	Gene product	191-202	471	N
LLLDVPTAAVQA	1210.704	-0.004	-3.11	2	1.76E-07	3.2	0.3	12643406	G-interferon-inducible lysosomal thiol reductase	26-37	261	N/I
RLAQEPLGLEVDQFLED	1972.002	0.002	1.21	2	1.30E-06	4.0	0.3	93141272	Glioma tumor suppressor	50-66	478	N
VNLPINGNGKQ	1153.632	-0.003	-2.84	2	2.24E-05	2.7	0.2	4504183	Glutathione transferase	200-210	210	N/I
LASPEYVNLPINGNGKQ	1813.944	0.003	1.76	2	4.28E-06	5.6	0.5	4504183	Glutathione transferase	194-210	210	N/I
ISWYDNEFGYSNRVVDL	2076.966	-0.004	-1.75	2	1.53E-07	4.1	0.4	7669492	Glyceraldehyde-3-phosphate dehydrogenase	311-327	335	N/I
INDPFIDLNY	1223.594	0.001	0.53	1	6.51E-04	2.3	0.2	7669492	Glyceraldehyde-3-phosphate dehydrogenase	33-42	335	N

HLA-A2 ligand sequence	Experimental mass [a]	ΔMass [b]	ΔMass [c]	z	P (pep) [d]	Xcorr [e]	ΔCn [e]	Gi accession	Protein	Position	Length of protein	Sample [f]
AAFNSGKVDIVAINDPFIDL	2119.107	0.002	0.81	2	1.65E-05	4.7	0.4	7669492	Glyceraldehyde-3-phosphate dehydrogenase	21-40	335	I
GLMTTVHAITATQK	1471.794	0.003	1.73	2	5.89E-08	3.3	0.4	7669492	Glyceraldehyde-3-phosphate dehydrogenase	173-186	335	N
SLMQAPLLIA	1056.612	-0.003	-2.55	1	7.92E-03	1.7	0.1	39995109	GM2 ganglioside activator	3-12	193	N
LLLAAPAQA	867.530	0.000	-0.23	1	7.01E-04	1.9	0.1	39995109	GM2 ganglioside activator	15-23	193	N/I
MLLGNPGLVFS	1147.618	-0.001	-0.72	2	9.70E-08	2.9	0.4	7108344	Granulysin isoform NKG5	12-22	145	N/I
SLYEGIDFYT	1207.552	0.001	0.80	1	4.69E-04	1.7	0.1	5729877	Heat shock 70kDa protein 8 isoform 1	286-295	646	N/I
SAVGFNEMEAPTTAYK	1715.795	-0.003	-1.76	2	6.41E-09	4.0	0.5	123557	Hematopoietic lineage cell-specific protein	208-223	486	N
GGNFGGRSSGPYGGGGQY	1674.726	-0.002	-1.01	2	4.87E-10	4.3	0.5	133254	Heterogeneous nuclear ribonucleoprotein A1	330-347	372	N
SSGSPYGGGYGSGGGSGGYGSR	1910.790	-0.002	-0.95	2	1.29E-08	4.9	0.5	34740329	Heterogeneous nuclear ribonucleoprotein A3	355-376	378	N
GPYGGGNYGPGGSGGSGGYGGRS	1974.833	-0.002	-1.02	2	5.33E-08	5.0	0.5	14043072	Heterogeneous nuclear ribonucleoprotein B1	329-351	353	N
SGNFGGSRNMGGP	1237.538	-0.001	-0.43	2	1.99E-03	3.0	0.4	14043072	Heterogeneous nuclear ribonucleoprotein B1	318-330	353	N
NMGGPYGGGNYGPGGSGGSGGYGGR	2189.905	0.000	0.01	2	8.66E-14	5.0	0.6	14043072	Heterogeneous nuclear ribonucleoprotein B1	326-350	353	N
YGGGNYGPGGSGGSGGYGGR	1733.726	0.001	0.36	2	2.43E-06	3.9	0.5	14043072	Heterogeneous nuclear ribonucleoprotein B1	331-350	353	N
TLPEVAECF	1008.471	-0.003	-3.37	1	8.82E-04	1.6	0.2	126302554	Heterogeneous nuclear ribonucleoprotein U	640-648	824	N
NQSQGYNQWQQGQFWGQKP	2309.048	-0.003	-1.22	2	3.77E-10	5.6	0.5	126302554	Heterogeneous nuclear ribonucleoprotein U	796-814	824	N
NQSQGYNQWQQGQF	1712.741	-0.002	-1.05	2	5.42E-06	4.0	0.4	126302554	Heterogeneous nuclear ribonucleoprotein U	796-809	824	N/I
PEVAECFDE	1038.408	0.001	1.42	1	1.13E-03	1.9	0.3	126302554	Heterogeneous nuclear ribonucleoprotein U	642-650	824	N
LLGGVTIAQGGVLPNIQAVLLPK	2271.380	0.004	1.80	2	7.50E-08	5.3	0.4	28195394	Histone cluster 2	97-119	130	I
GIAEEPEIQMV	1215.593	-0.001	-0.97	1	2.50E-04	1.7	0.1	15277507	Histone H4 transcription factor	507-517	517	N
IAVGYVDDTQFVRFDSD	1946.913	0.002	1.08	2	6.17E-12	4.8	0.5	717123	HLA-A2	47-63	365	N/I
APWIEQEGPEYWDGETR	2062.914	-0.005	-2.37	2	4.55E-14	6.0	0.4	717123	HLA-A2	73-89	365	N/I
IAVGYVDDTQF	1227.589	-0.005	-3.91	1	6.22E-07	2.4	0.4	717123	HLA-A2	47-57	365	N/I
YAYDGKDYIAL	1291.620	-0.004	-2.96	2	1.92E-03	2.6	0.2	717123	HLA-A2	140-150	365	N/I
IAVGYVDDTQFVRF	1629.827	-0.003	-1.61	2	1.81E-09	4.1	0.4	717123	HLA-A2	47-60	365	N/I
IAVGYVDDTQ	1080.521	-0.002	-1.78	1	1.10E-04	2.2	0.3	717123	HLA-A2	47-56	365	N
VGYVDDTQF	1043.468	0.000	-0.35	1	9.57E-03	1.7	0.2	717123	HLA-A2 [g]	49-57	365	N/I
FLLPLVMQGVSRA	1430.819	0.001	0.54	2	5.17E-04	2.5	0.2	4557880	Interferon gamma receptor 1	5-17	489	N
FLLPLVMQGVSRAEM	1690.902	0.003	1.66	2	7.52E-05	3.7	0.4	4557880	Interferon gamma receptor 1	5-19	489	N/I
SLQELQGQDPTHLV	1564.797	-0.005	-3.43	2	2.73E-08	4.2	0.4	4504665	Interleukin 2 receptor	538-551	551	N
ALYEDGALDSLQLL	1520.784	0.000	0.03	2	7.19E-04	2.9	0.3	68800243	Interleukin-1 receptor-associated kinase 1	674-687	712	N
FIFQQPEAPMEGFQL	1781.857	0.001	0.68	2	2.13E-06	2.9	0.3	6912478	Karyopherin alpha 6	522-536	536	N
SADTLWGIQKELQF	1635.838	-0.001	-0.67	2	2.04E-04	2.9	0.3	5031857	Lactate Dehydrogenase A	319-332	332	N/I
TLWDIQKDLKDL	1487.810	-0.002	-1.68	2	1.03E-05	3.3	0.2	4557032	Lactate Dehydrogenase B	323-334	334	N/I
LLWVPGCFA	1005.523	0.000	0.42	1	7.53E-05	1.7	0.3	56550061	Leukocyte membrane antigen	9-17	299	N
FLGPWPAAS	945.483	-0.001	-1.32	1	6.21E-04	1.7	0.1	4505021	Low density lipoprotein receptor	22-30	357	N/I
FLYPLVGTMS	1127.581	-0.001	-1.21	1	1.24E-04	1.8	0.3	115206	Methylenetetrahydrofolate dehydrogenase	899-908	935	N
SMFDQSQIQEFK	1487.684	-0.001	-0.42	2	2.26E-06	4.1	0.4	284326	Myosin regulatory light chain 2	57-68	204	N
AMFDQSQIQEFK	1471.689	-0.005	-3.60	2	8.77E-06	3.4	0.3	15809016	Myosin regulatory light chain MRCL2	24-35	172	N
ATSNVFAMFDQSQIQEFK	2090.985	-0.004	-1.75	2	1.79E-09	5.7	0.5	15809016	Myosin regulatory light chain MRCL2	18-35	172	N
EEVGEEAIVELVENGK	1743.865	-0.003	-1.80	2	4.76E-07	4.5	0.3	12667788	Myosin, heavy polypeptide 9, non-muscle	48-63	1960	N
KVKVNKDDIQK	1314.774	-0.003	-1.95	2	5.51E-04	3.9	0.2	12667788	Myosin, heavy polypeptide 9, non-muscle	64-74	1960	N
GKADGAEAKPAE	1143.564	-0.001	-1.15	2	1.56E-05	3.9	0.3	12667788	Myosin, heavy polypeptide 9, non-muscle	1949-1960	1960	N
VKVNKDDIQK	1186.679	-0.001	-0.92	2	3.49E-03	2.9	0.2	12667788	Myosin, heavy polypeptide 9, non-muscle	65-74	1960	N
SGFEPASLKEEVGEEAIVEL	2133.060	0.004	1.90	2	3.74E-07	4.5	0.3	12667788	Myosin, heavy polypeptide 9, non-muscle	39-58	1960	N
LLADIGGDPFA	1088.562	-0.004	-3.37	1	8.58E-04	1.9	0.2	3135319	Nucleoporin	151-161	318	N
VLLAAGPSAA	869.509	-0.001	-1.68	1	7.49E-05	2.0	0.4	27477134	Nucleoporin 210	15-24	1887	N/I
VLPGVDALSNI	1097.620	-0.004	-3.95	2	2.33E-03	2.6	0.3	4505763	Phosphoglycerate kinase 1	407-417	417	N/I

HLA-A2 ligand sequence	Experimental mass [a]	∆Mass [b]	∆Mass [c]	z	P (pep) [d]	Xcorr [e]	∆Cn [e]	Gi accession	Protein	Position	Length of protein	Sample [f]
YQYDPEQLDLAENMVSQNDGSF	2563.093	0.002	0.90	2	2.12E-10	5.4	0.5	42560237	Poliovirus receptor-related 1	488-509	517	N
SLLQDGEFSM	1126.509	-0.001	-1.23	1	1.65E-06	2.1	0.1	4826898	Profilin 1	77-86	140	N
PVDLSKWSGPLSL	1398.763	0.001	0.96	2	4.24E-05	3.8	0.4	4505621	Prostatic binding protein	2-14	187	N
NLQVTQPTV	999.547	-0.002	-2.26	1	7.62E-04	2.3	0.3	4506693	Ribosomal protein S17	116-124	135	N
FSLPIKESEIIDF	1537.815	0.001	0.85	2	5.04E-04	3.2	0.5	15055539	Ribosomal protein S2	84-96	293	N/I
VFEVSLADLQNDEVAFR	1951.976	-0.005	-2.64	2	5.39E-07	4.3	0.5	4506723	Ribosomal protein S3a	66-82	264	N
VLEGKELEFYL	1339.714	-0.004	-2.65	2	5.77E-03	2.6	0.2	4506743	Ribosomal protein S8	189-199	208	N/I
YVLEGKELEFYL	1502.778	0.000	-0.02	2	5.46E-05	2.9	0.3	4506743	Ribosomal protein S8	188-199	208	N/I
VLLESEQFLTELTRL	1790.990	-0.005	-3.04	2	8.47E-08	3.9	0.5	149999611	Signal recognition particle 14kDa	2-16	136	N
VQGLGEGVSTDQVGEF	1621.770	-0.003	-1.82	2	1.11E-08	3.7	0.4	21327701	TBP-associated factor 15	238-253	592	N
LLLAQLSDA	943.546	-0.003	-3.39	1	2.60E-04	1.9	0.4	11034855	TMEM9 domain family	25-33	198	N/I
NLFGGEPLSYT	1197.579	-0.002	-1.92	1	3.96E-03	2.5	0.4	189458817	Transferrin receptor	11-21	760	N
FLYPFPLAL	1080.613	0.001	0.71	1	1.29E-04	1.5	0.2	66392190	Transmembrane protein 41B	141-149	291	N
VDISQQYSNTQTFTGK	1816.871	0.001	0.69	2	7.69E-09	4.6	0.5	20162566	TTD non-photosensitive 1 protein	158-173	179	N
VDLEPTVIDEVRTGTY	1806.912	-0.003	-1.72	2	1.48E-09	4.5	0.4	14389309	Tubulin alpha 6	68-83	449	N
TIGGGDDSFNTFFSETGAGK	2007.893	0.000	0.09	2	1.68E-09	5.4	0.6	14389309	Tubulin alpha 6	41-60	449	N
ILAPAGSLPKI	1079.682	-0.003	-2.59	2	6.84E-04	2.8	0.3	7019551	UbiA prenyltransferase domain	328-338	338	N/I
RLFQKLEENGDLYLAV	1908.023	-0.001	-0.37	2	3.06E-05	2.7	0.2	42716287	WD repeat domain 41	444-459	459	N

[a] Mass of monoisotopic ion in amu

[b] Difference between nominal and experimentally detected monoisotopic ions in amu

[c] Difference between nominal and experimentally detected monoisotopic ions in ppm

[d] Probability of finding a match better than the observed match by chance

[e] Sequest score function

[f] N: non-infected; I: infected

[g] All common peptides between HLA class I molecules were asigned as HLA-A2
