# Supplementary material for: Diversity of Natural Self-Derived Ligands Presented by Different HLA Class I Molecules in Transporter Antigen Processing-Deficient Cells
Source: PLoS One. 2013 Mar 26;8(3):e59118. doi: 10.1371/journal.pone.0059118 (PMC3608615; doi:10.1371/journal.pone.0059118)
Supplement: Table S2 — Summary of HLA-B27 ligands identified by mass spectrometry analysis. (PDF) [file pone.0059118.s009.pdf]

Supplemental Table 2. Summary of HLA-B27 ligands identified by mass spectrometry analysis

HLA-B27 ligand sequence	Experimental mass [a]	ΔMass [b]	ΔMass [c]	z	P (pep) [d]	Xcorr [e]	ΔCn [e]	Gi accession	Protein	Position	Length of protein	Sample [f]
ASSEGGTAAGAGLDSLHK	1628.787	-0.001	-0.64	2	1.11E-07	4.6	0.5	5031601	Actin related protein	309-326	372	N
GQYGNPLNKY	1153.564	-0.002	-1.48	2	3.18E-03	2.5	0.3	4557251	ADAM metallopeptidase domain 10	19-28	748	I
AGFAGDDAPR	976.448	-0.001	-1.44	2	9.91E-06	3.0	0.4	4501885	Beta Actin	19-28	375	N
DESGPSIVHRKCF	1474.711	-0.003	-2.33	2	1.62E-05	2.6	0.2	4501885	Beta Actin	363-375	375	N
DLYANTVLSGGTTMYPGIADRMQK	2602.264	-0.003	-1.32	3	6.36E-09	4.8	0.5	4501885	Beta Actin	292-315	375	N
LVVDNGSGMCK	1122.528	0.000	-0.05	2	3.23E-05	3.0	0.3	4501885	Beta Actin	8-18	375	N
LVVDNGSGMCKAGFAGDDAPRAVF	2397.133	-0.003	-1.13	3	4.44E-15	4.8	0.5	4501885	Beta Actin	8-31	375	N
MGQKDSYVGDEAQSK	1642.738	0.002	1.49	2	6.96E-09	4.8	0.6	4501885	Beta Actin	47-61	375	N
YNELRVAPEEHPVL	1665.859	-0.003	-1.74	3	7.22E-08	3.8	0.2	4501885	Beta Actin	91-104	375	N/I
HDGHDDDVIDIEDDL	1722.709	0.001	0.51	2	5.98E-07	3.6	0.4	10716563	Calnexin precursor	21-35	592	N/I
HDGHDDDVIDIEDDLDD	1952.763	0.000	-0.09	2	2.64E-08	4.1	0.4	10716563	Calnexin precursor	21-37	592	N/I
WQVKSGTIFDNF	1441.711	-0.004	-2.67	2	5.36E-06	2.8	0.4	4757900	Calreticulin precursor	319-330	417	I
PSSGLGVTKQDLGPVPM	1682.878	0.001	0.70	2	6.38E-07	4.0	0.4	68448544	CD74a	280-296	296	N/I
ASMQQQQQLASAR	1446.712	-0.002	-1.30	2	6.22E-07	3.3	0.4	119573678	Chromosome 1 open reading frame 77	39-51	154	N
GRLLVVYPWTQ	1331.747	-0.004	-3.36	2	1.91E-07	3.1	0.3	4504351	Delta globin	30-40	147	N/I
APGSVTSRLGSVFPF	1521.806	-0.001	-0.69	2	9.65E-09	2.7	0.4	5453980	DnaJ (Hsp40) homolog	3-17	504	N/I
VQGLNDSVTLDDLADF	1721.823	-0.001	-0.69	2	6.69E-10	3.8	0.4	4885225	Ewing sarcoma	365-380	656	N/I
VQGLGENVTIESVADY	1693.828	-0.004	-2.60	2	7.16E-07	3.6	0.5	4826734	Fusion	289-304	526	I
ISWYDNEFGYSNRVVDL	2076.966	-0.004	-1.86	2	3.73E-12	5.3	0.5	7669492	Glyceraldehyde-3-phosphate dehydrogenase	311-327	335	I
ALSRQEMQEVQSSR	1648.807	-0.003	-2.02	2	1.21E-04	3.5	0.1	14043072	Heterogeneous nuclear ribonucleoprotein B1	187-200	353	N
GAAKEAAGKSSGPTSL	1431.744	-0.001	-0.53	2	1.56E-06	3.6	0.3	126302554	Heterogeneous nuclear ribonucleoprotein U	178-193	824	N/I
NQSQGYNQWQQGQF	1712.741	-0.004	-2.47	2	3.40E-07	4.2	0.4	126302554	Heterogeneous nuclear ribonucleoprotein U	796-809	824	N
VEGKDLPEHAVLK	1434.795	-0.002	-1.10	2	9.07E-07	3.8	0.4	126302554	Heterogeneous nuclear ribonucleoprotein U	622-634	824	N
GRSSGPYGGGGQYF	1389.618	-0.003	-2.11	2	1.93E-06	3.8	0.5	133254	Heterogeneous ribonucleoprotein A1	335-348	372	N
ITLTWQRDGEDQTQDTEL	2149.004	-0.003	-1.16	2	2.14E-06	4.1	0.4	717123	HLA-A2	237-254	365	I
LRWEPSSQPTIPIVG	1679.912	0.002	1.44	2	2.71E-04	2.8	0.1	717123	HLA-A2	296-310	365	N/I
HRVDLGTLR	1066.612	0.000	-0.15	3	1.40E-05	3.7	0.4	717123	HLA-A2 [g]	98-106	365	N/I
SWTAADTAAQITQR	1519.750	-0.002	-1.03	2	1.81E-09	4.4	0.4	32177	HLA-B27	156-169	362	N
DIVLTQSPASL	1143.626	-0.002	-1.88	2	4.49E-04	3.4	0.1	418845	Ig kappa chain precursor	21-31	140	N/I
DIVLTQSPASLAVSLGQ	1698.927	-0.003	-1.76	2	2.55E-09	4.6	0.5	418845	Ig kappa chain precursor	21-37	140	I
DIVLTQSPASLAVSLGQR	1855.028	-0.004	-1.98	2	1.83E-11	4.9	0.5	418845	Ig kappa chain precursor	21-38	140	N/I
DIVLTQSPASLAVSLGQRA	1926.065	0.000	-0.13	2	2.34E-13	5.7	0.5	418845	Ig kappa chain precursor	21-39	140	I
ASYTWSDAAAAFAGLSREE	2002.914	-0.004	-2.01	2	9.99E-11	5.3	0.6	23821023	Interleukin 4 induced 1	396-414	567	I
NADHSMNYQYR	1398.586	0.000	-0.16	2	3.96E-07	2.9	0.6	24234750	Interleukin enhancer-binding factor 3	884-894	894	N
YRSQQEAAAKKFF	1573.812	-0.001	-0.94	3	4.37E-08	3.5	0.2	5031873	Lectin, mannose-binding, 1 precursor	498-510	510	N/I
EALSLPSKTPEGGPAPPPYSEV	2223.118	-0.001	-0.66	2	1.00E-11	4.5	0.5	5803056	Lysosomal multispanning membrane protein 5	241-262	262	N/I
EEALSLPSKTPEGGPAPPPYSEV	2352.161	-0.004	-1.62	2	2.07E-11	5.6	0.5	5803056	Lysosomal multispanning membrane protein 5	240-262	262	N/I
KTPEGGPAPPPYSEV	1525.753	-0.005	-3.15	2	2.74E-08	4.5	0.5	5803056	Lysosomal multispanning membrane protein 5	248-262	262	N/I
LPSKTPEGGPAPPPYSEV	1822.922	-0.004	-2.08	2	1.07E-13	4.4	0.5	5803056	Lysosomal multispanning membrane protein 5	245-262	262	N/I
PSYEEALSLPSKTPEGGPAPPPYSEV	2699.309	-0.005	-1.80	2	3.53E-11	5.2	0.5	5803056	Lysosomal multispanning membrane protein 5	237-262	262	N/I
SKTPEGGPAPPPYSEV	1612.785	0.001	0.46	2	1.24E-10	4.4	0.5	5803056	Lysosomal multispanning membrane protein 5	247-262	262	N/I
SLPSKTPEGGPAPPPYSEV	1909.954	0.002	0.98	2	2.50E-07	4.1	0.5	5803056	Lysosomal multispanning membrane protein 5	244-262	262	N/I
TPEGGPAPPPYSEV	1397.658	0.000	-0.20	2	2.63E-06	3.0	0.4	5803056	Lysosomal multispanning membrane protein 5	249-262	262	N/I
FRLFPVPGSGLV	1288.741	0.001	1.11	2	3.23E-07	2.7	0.5	4504957	Lysosomal-associated membrane protein 2	4-15	410	I
AMFDQSQIQEFK	1471.689	-0.001	-0.78	2	1.39E-05	3.4	0.4	15809016	Myosin regulatory light chain MRCL2	24-35	172	N
AMFDQSQIQEFKEAF	1818.837	0.001	0.47	2	2.44E-06	3.1	0.5	15809016	Myosin regulatory light chain MRCL2	24-38	172	N/I
FDQSQIQEFK	1269.611	0.000	-0.11	2	7.26E-05	2.9	0.3	15809016	Myosin regulatory light chain MRCL2	26-35	172	N

HLA-B27 ligand sequence	Experimental mass [a]	ΔMass [b]	ΔMass [c]	z	P (pep) [d]	Xcorr [e]	ΔCn [e]	Gi accession	Protein	Position	Length of protein	Sample [f]
KGNFNYIEFTR	1388.696	-0.001	-1.06	2	1.38E-07	3.4	0.2	15809016	Myosin regulatory light chain MRCL2	151-161	172	N
PEDVIRNAF	1060.542	0.000	-0.09	2	1.38E-05	2.6	0.1	15809016	Myosin regulatory light chain MRCL2	99-107	172	I
TRILKHGAKDKDD	1496.818	0.000	-0.05	2	2.21E-06	3.5	0.5	15809016	Myosin regulatory light chain MRCL2	160-172	172	N/I
AGDGSDEEVDGKADGAEAKPAE	2117.911	0.005	2.56	3	9.95E-12	5.1	0.3	12667788	Myosin, heavy polypeptide 9, non-muscle	1939-1960	1960	N
EVDGKADGAEAKPAE	1486.702	-0.001	-0.88	2	1.22E-04	3.6	0.3	12667788	Myosin, heavy polypeptide 9, non-muscle	1946-1960	1960	N
GAGDGSDEEVDGKADGAEAKPAE	2174.932	0.001	0.37	2	9.90E-09	5.3	0.4	12667788	Myosin, heavy polypeptide 9, non-muscle	1938-1960	1960	N
KLVWVPSDKSGFEPASLK	1988.085	0.000	-0.02	3	4.36E-07	4.7	0.6	12667788	Myosin, heavy polypeptide 9, non-muscle	30-47	1960	N
NFINNPLAQADWAAK	1672.844	0.001	0.74	2	1.45E-07	5.2	0.4	12667788	Myosin, heavy polypeptide 9, non-muscle	15-29	1960	N
NTDQASMPDNTAAQK	1591.702	0.003	1.67	2	8.88E-05	4.6	0.4	12667788	Myosin, heavy polypeptide 9, non-muscle	359-373	1960	N
RGDLPFVVPR	1155.663	0.001	0.67	2	2.06E-05	3.4	0.3	12667788	Myosin, heavy polypeptide 9, non-muscle	1923-1932	1960	N
RHEMPPHIYAITDTAYR	2071.018	-0.005	-2.41	3	9.81E-12	4.9	0.5	12667788	Myosin, heavy polypeptide 9, non-muscle	143-159	1960	N
VIQYLAYVASSHK	1478.800	0.002	1.05	2	1.94E-06	3.7	0.4	12667788	Myosin, heavy polypeptide 9, non-muscle	187-199	1960	N
YLYVDKNFINNPLAQADWAAK	2454.245	-0.003	-1.09	3	1.67E-10	6.4	0.5	12667788	Myosin, heavy polypeptide 9, non-muscle	9-29	1960	N
VSWADGGQHSSIHPA	1548.719	-0.001	-0.59	2	3.77E-06	3.3	0.4	4826862	Nucleoside-diphosphate kinase 4	173-187	187	N/I
LLTTTPRPVIVEPMEQ	1823.994	0.000	-0.08	2	5.84E-07	3.7	0.4	109240550	Paraspeckle protein 1	222-237	523	I
LLTTTPRPVIVEPMEQF	1971.062	0.002	0.94	2	1.04E-06	3.5	0.3	109240550	Paraspeckle protein 1	222-238	523	I
IDLSDVELDDLGKDEL	1788.875	-0.005	-2.59	2	1.52E-05	5.2	0.3	119621354	Protein disulfide isomerase	244-259	259	I
KRIALTDNALIAR	1454.880	-0.004	-2.69	2	9.54E-10	3.6	0.2	15431301	Ribosomal protein L7	165-177	248	I
KRVLLGETGKEKLPR	1724.054	-0.004	-2.19	3	4.77E-05	4.4	0.3	4506681	Ribosomal protein S11	21-35	158	I
VRMNVLADALK	1229.703	-0.002	-1.66	2	2.17E-05	3.1	0.2	14165469	Ribosomal protein S15a	2-12	130	I
RRLPDAHSDYAR	1456.740	0.002	1.16	2	2.37E-04	2.6	0.3	5454064	RNA binding motif protein 14	636-647	669	I
SRWNQDTMEQK	1422.643	-0.003	-2.20	2	7.47E-04	3.0	0.3	119594698	Splicing factor 1	20-30	587	N
INKGKGFGF	967.536	-0.005	-5.15	2	7.37E-04	2.5	0.2	4826998	Splicing factor proline/glutamine rich	328-336	707	N
QTNNQNWGSQPIAQQPL	1923.931	0.000	-0.23	2	1.23E-05	4.0	0.4	119569012	Synaptotagmin binding	544-560	588	N
FYVPGVAPINFHQND	1717.833	0.003	1.91	2	3.97E-05	2.7	0.4	164519076	Transmembrane 9 protein	24-38	642	N/I
VDISQQYSNTQTFTGK	1816.871	-0.005	-2.66	2	2.11E-06	4.7	0.5	20162566	TTD non-photosensitive 1 protein	158-173	179	N/I
TIGGGDDSFNTFFSETGAGK	2007.893	-0.003	-1.31	2	1.06E-09	5.7	0.6	14389309	Tubulin alpha 6	41-60	449	N
VDLEPTVIDEVRTGTY	1806.912	-0.002	-1.38	2	4.33E-09	4.7	0.5	14389309	Tubulin alpha 6	68-83	449	N/I
TTFPRPVTVEPMDQL	1730.878	-0.005	-2.77	2	4.87E-05	3.1	0.2	348239	Unnamed protein product	216-230	471	I
FGGPGTASRPSSS	1207.570	0.003	2.18	2	2.13E-07	2.5	0.3	62414289	Vimentin	15-27	466	N

[a] Mass of monoisotopic ion in amu

[b] Difference between nominal and experimentally detected monoisotopic ions in amu

[c] Difference between nominal and experimentally detected monoisotopic ions in ppm

[d] Probability of finding a match better than the observed match by chance

[e] Sequest score function

[f] N: non-infected; I: infected

[g] All common peptides between HLA class I molecules were asigned as HLA-A2
