# Supplementary material for: Diversity of Natural Self-Derived Ligands Presented by Different HLA Class I Molecules in Transporter Antigen Processing-Deficient Cells
Source: PLoS One. 2013 Mar 26;8(3):e59118. doi: 10.1371/journal.pone.0059118 (PMC3608615; doi:10.1371/journal.pone.0059118)
Supplement: Table S3 — Summary of HLA-B51, or -Cw1 ligands identified by mass spectrometry analysis. (PDF) [file pone.0059118.s010.pdf]

Supplemental Table 3. Summary of HLA-B51, or -Cw1 ligands identified by mass spectrometry analysis

HLA-B51, or -Cw1 ligand sequence	Experimental mass [a]	ΔMass [b]	ΔMass [c]	z	P (pep) [d]	Xcorr [e]	ΔCn [e]	Gi accession	Protein	Position	Length of protein	Sample [f]
LPNHRVLIM	1092.635	0.001	0.63	2	5.19E-04	3.0	0.3	30047108	ABHD14B protein	203-211	238	NI
SQIDDLYSTIKV	1381.721	0.000	-0.22	2	4.41E-06	3.7	0.3	40807491	ACSL1	687-698	698	NI
LPYKVADIGL	1088.635	-0.001	-1.04	2	1.27E-03	3.3	0.2	9951915	Adenosylhomocysteinase	5-14	432	NI/I
LPNWQGSGSHGLTI	1466.739	-0.001	-0.69	2	3.14E-03	2.5	0.2	15214688	AHNAK nucleoprotein	12-25	149	NI
ERMFLSFPTTK	1356.698	-0.004	-2.80	2	8.23E-04	2.9	0.3	4504345	Alpha 2 globin	31-41	142	NI
YPYWPHQPIE	1329.626	-0.001	-1.02	2	6.26E-05	2.8	0.2	5453559	ATP synthase	150-159	161	NI/I
NKELDPIQKLF	1344.752	0.000	-0.14	2	1.88E-04	3.5	0.2	18644883	ATP synthase-coupling factor 6	33-43	108	NI
PKFEVIEKPQA	1285.715	0.000	-0.25	2	7.90E-05	3.3	0.3	18644883	ATP synthase-coupling factor 6	98-108	108	NI/I
YLDPALELGPRNV	1456.779	0.003	1.84	2	3.59E-06	3.5	0.4	17136148	ATPase	77-89	470	I
LMLEEVTGLK	1132.628	0.000	-0.21	2	3.60E-04	2.7	0.3	187611510	ATPase family protein	281-290	893	NI
LPYPAPEGI	956.509	0.000	-0.41	1	1.22E-04	1.7	0.1	183396787	BCL-6 corepressor	658-666	1755	NI
YTEFTPTEKDE	1359.595	0.003	2.55	2	7.59E-04	3.0	0.4	114319011	Beta 2-microglobulin	87-97	124	I
AGFAGDDAPRAVF	1293.622	-0.001	-1.14	2	2.34E-05	3.1	0.4	4501885	Beta Actin	19-31	375	NI
AGFAGDDAPRAVFPSIVGRPR	2156.136	-0.004	-1.93	3	3.07E-11	5.0	0.5	4501885	Beta Actin	19-39	375	NI
DESGPSIVHRKCF	1474.711	-0.003	-1.75	2	2.25E-10	3.3	0.3	4501885	Beta Actin	363-375	375	NI
DFEQEMATAASSSSLEKS	1917.838	-0.001	-0.29	2	1.16E-07	4.7	0.4	4501885	Beta Actin	222-239	375	NI
DLYANTVLSGGTTMYPGIADR	2215.070	0.004	2.02	2	1.14E-07	5.0	0.5	4501885	Beta Actin	292-312	375	NI
LRVAPEEHPVL	1259.711	0.000	0.02	2	1.63E-06	2.5	0.3	4501885	Beta Actin	94-104	375	NI/I
LRVAPEEHPVLLTEAPLNPK	2223.250	-0.004	-1.97	2	1.50E-10	6.0	0.3	4501885	Beta Actin	94-113	375	NI/I
LTEAPLNPKANREKMTQ	1941.022	-0.001	-0.59	2	1.31E-07	3.7	0.5	4501885	Beta Actin	105-121	375	NI
LVVDNGSGMCKAGFAGD	1640.741	-0.002	-0.93	2	1.31E-09	4.3	0.6	4501885	Beta Actin	8-24	375	NI
MQKEITALAPSTMK	1548.812	0.000	-0.17	2	1.78E-06	4.1	0.3	4501885	Beta Actin	313-326	375	NI
YASGRTTGIVMDSGD	1529.690	-0.003	-1.64	2	1.80E-07	2.9	0.3	4501885	Beta Actin	143-157	375	NI
YDESGPSIVHRKCF	1637.774	-0.002	-1.00	2	2.04E-08	3.4	0.4	4501885	Beta Actin	362-375	375	NI
YNELRVAPEEHPVL	1665.859	-0.002	-1.13	2	2.87E-07	3.1	0.4	4501885	Beta Actin	91-104	375	NI/I
YNELRVAPEEHPVLL	1778.944	-0.001	-0.53	2	4.05E-05	2.6	0.2	4501885	Beta Actin	91-105	375	NI
MSMKEVDEQMLNVQNK	1923.897	0.000	0.20	2	3.99E-07	5.3	0.4	2119276	Beta tubulin	314-329	437	NI
YPPYPPQPM	1089.507	0.001	0.84	1	8.08E-07	2.3	0.2	18645165	C5orf32 protein	18-26	97	NI
APSRQDVYGPQPQV	1541.771	-0.002	-1.11	2	2.49E-08	3.4	0.3	146231940	Catenin	250-263	968	NI/I
EDPSSGLGVTKQDLGPVPM	1926.948	-0.001	-0.42	2	3.56E-09	4.8	0.4	68448544	CD74a	278-296	296	NI
ELEDPSSGLGVTKQDLGPVPM	2169.074	0.003	1.18	2	3.27E-06	4.5	0.4	68448544	CD74a	276-296	296	NI
LEDPSSGLGVTKQDLGPVPM	2040.032	0.004	1.91	2	3.07E-06	4.4	0.3	68448544	CD74a	277-296	296	NI
MRMATPLLMQALPMG	1660.841	0.000	0.11	2	1.31E-04	2.9	0.3	68448544	CD74a	107-121	296	NI
FSAPLPLENETEISESGMTVR	2307.117	0.001	0.45	2	1.44E-14	4.9	0.5	109255228	Centrosomal protein 170kDa	606-626	1584	NI
FLPTPEELGLLGPPRPQVLA	2144.211	0.004	2.02	2	4.52E-11	4.6	0.5	4759328	Class I cytokine receptor	617-636	636	NI/I
GVIKVFNDM	1022.534	0.000	-0.01	1	8.26E-03	1.6	0.2	5031635	Cofilin 1	10-18	166	NI
SSGGSYRDSYDSYATHNE	1995.795	-0.005	-2.56	2	1.20E-06	3.8	0.5	4502847	Cold inducible RNA binding protein	155-172	172	NI
SLWGQPAEA	958.463	0.001	0.55	1	1.94E-04	1.6	0.3	156230677	Collagen, type IV, alpha 5	18-26	1691	NI/I
DVKFGADARALMLQGVDL	1919.006	0.002	1.00	2	1.37E-08	4.2	0.4	31542947	Chaperonin	29-46	573	NI/I
FGADARALMLQGVDL	1576.815	0.000	-0.18	2	4.47E-05	3.5	0.2	31542947	Chaperonin	32-46	573	NI
LHLGYLPNQLFRTF	1718.938	-0.001	-0.84	2	3.43E-11	3.9	0.4	4826686	DEAD box polypeptide 1	727-740	740	NI
NPYDRGSAFAIGSDG	1526.687	0.000	-0.03	2	7.53E-08	2.8	0.3	4826686	DEAD box polypeptide 1	94-108	740	I
TLDEKIEKV	1074.604	-0.001	-1.14	1	5.41E-04	2.0	0.1	119596071	DEAD box polypeptide 27	85-93	440	NI
SREDAGDNDDTEGAIGVRNK	2118.965	0.005	2.41	3	9.81E-09	6.0	0.4	20149629	DEAD box polypeptide 47	424-443	455	NI
HRLDLGEDYPSGK	1486.728	-0.001	-0.79	2	2.39E-05	3.6	0.4	68509926	DEAH box polypeptide 15	5-17	795	NI
FPFPTPPSV	988.514	0.001	1.22	1	3.11E-04	2.0	0.2	29029601	DEAH box polypeptide 37	723-731	1157	NI

HLA-B51, or -Cw1 ligand sequence	Experimental mass [a]	ΔMass [b]	ΔMass [c]	z	P (pep) [d]	Xcorr [e]	ΔCn [e]	Gi accession	Protein	Position	Length of protein	Sample [f]
KQIPDSLSTDL	1216.642	-0.002	-1.65	2	5.40E-05	2.6	0.2	31377468	Dedicator of cytokinesis 2	1820-1830	1830	NI
KRSSVVFADEKAAAESDLK	2051.077	-0.004	-1.96	3	2.90E-06	5.0	0.3	31377468	Dedicator of cytokinesis 2	1703-1721	1830	NI
LHVDPENFRLLGNVL	1735.949	-0.003	-1.71	2	1.35E-07	3.1	0.3	4504351	Delta globin	97-111	147	NI
LVVYPWTQRF	1308.710	-0.001	-0.72	2	3.30E-06	2.7	0.3	4504351	Delta globin	33-42	147	NI
GAVHDVKDVLDS	1254.632	0.002	1.92	2	1.97E-04	2.9	0.2	16751921	Dermcidin	97-108	110	I
DPFNPFELTNHAV	1500.712	0.005	3.37	2	8.56E-09	3.6	0.2	189083844	Dipeptidyl peptidase 1	395-407	463	NI/I
APGSVTSRLGSVFPF	1521.806	0.000	-0.13	2	2.40E-07	2.8	0.4	5453980	DnaJ (Hsp40) homolog	3-17	504	NI
AAFKELQSTFK	1269.684	-0.001	-0.48	2	1.56E-05	2.8	0.3	20149675	EF-hand domain D2	230-240	240	NI
KYEEIDNAPEER	1492.691	0.004	2.52	2	9.63E-09	4.0	0.4	1706611	Elongation factor Tu	91-102	452	NI
LRIEEELGSK	1173.647	-0.002	-2.09	2	1.30E-04	2.9	0.1	4503571	Enolase 1	411-420	434	NI
ALPGDNVGFNV	1102.553	0.001	0.46	1	3.26E-03	1.7	0.3	4503471	Eukaryotic translation elongation factor 1 alpha 1	302-312	462	NI
VQSMDVAAFNKI	1322.677	0.000	-0.09	2	5.48E-04	2.8	0.3	4503477	Eukaryotic translation elongation factor 1 beta 2	214-225	225	NI/I
ITEHGDLGNSRFLD	1573.761	-0.001	-0.95	2	4.50E-08	2.9	0.4	62898013	F-actin capping protein	76-89	286	NI
SYKIGKEMQNA	1268.630	-0.002	-1.88	2	6.32E-06	2.5	0.3	62898013	F-actin capping protein	276-286	286	NI
LPPGPGGTM	826.413	0.000	0.41	1	2.72E-04	1.9	0.1	29789054	FCH domain only 1 [Homo sapiens]	389-397	889	NI
ALSDLEITLEGGK	1345.721	0.000	-0.04	2	2.78E-06	3.4	0.3	29789006	Fermitin family homolog 2	349-361	680	NI
AQTSPQGMPQHPPAPQGQ	1856.871	-0.003	-1.43	2	8.65E-06	3.7	0.4	17402900	FUSE-binding protein 1	627-644	644	NI/I
YGQTLGQAQAHSQEQ	1645.756	0.004	2.50	2	2.02E-08	4.1	0.4	100816392	FUSE-binding protein 3	558-572	572	NI/I
LLLDVPTAAVQA	1210.704	-0.003	-2.31	2	2.57E-06	3.5	0.4	12643406	G-interferon-inducible lysosomal thiol reductase	26-37	261	NI/I
RLAQEPLGLEVDQF	1614.849	0.001	0.63	2	6.84E-07	3.6	0.4	93141272	Glioma tumor suppressor	50-63	478	NI
INDPFIDLNY	1223.594	0.005	4.32	1	7.71E-04	2.5	0.2	7669492	Glyceraldehyde-3-phosphate dehydrogenase	33-42	335	I
PSKIKWGDAGAEY	1421.706	0.002	1.61	2	3.44E-06	3.2	0.3	7669492	Glyceraldehyde-3-phosphate dehydrogenase	82-94	335	I
GLMTTVHAITATQK	1471.794	0.001	0.40	2	2.67E-06	3.8	0.4	7669492	Glyceraldehyde-3-phosphate dehydrogenase	173-186	335	NI
VVESTGVFTTMEK	1427.709	-0.002	-1.46	2	5.93E-09	2.8	0.5	7669492	Glyceraldehyde-3-phosphate dehydrogenase	95-107	335	NI
FGPEWLRAL	1088.589	-0.002	-1.39	2	9.33E-05	2.7	0.2	42476299	GRB10 interacting GYF protein	10-18	1299	NI
VPFERPAVI	1027.593	-0.001	-0.77	2	4.83E-04	2.6	0.4	119220596	Helios	258-266	526	NI
ETTDESLRSHFEQWGTLTD	2252.010	0.001	0.60	2	1.81E-09	3.7	0.3	133254	Heterogeneous nuclear ribonucleoprotein A1	24-42	372	NI
GGNFGGRSSGPYGGGGQY	1674.726	-0.003	-1.82	2	4.07E-08	4.9	0.6	133254	Heterogeneous nuclear ribonucleoprotein A1	330-347	372	NI
NQGGYGGSSSSSSYGSGR	1694.700	-0.003	-1.60	2	2.78E-09	4.8	0.6	133254	Heterogeneous nuclear ribonucleoprotein A1	353-370	372	NI
SSGPYGGGGQYFAKPR	1628.782	-0.002	-1.25	2	1.09E-08	3.7	0.5	133254	Heterogeneous nuclear ribonucleoprotein A1	337-352	372	NI
RQTNNQQNWGSQPIAQQPL	2208.090	0.003	1.25	2	3.92E-14	4.8	0.5	5031755	Heterogeneous nuclear ribonucleoprotein R	586-604	633	NI
YQDTYGQQWK	1316.591	-0.002	-1.32	2	2.40E-04	3.2	0.4	5031755	Heterogeneous nuclear ribonucleoprotein R	624-633	633	NI/I
GYFEYIEENKYSR	1697.781	-0.001	-0.57	2	2.42E-07	3.9	0.4	126302554	Heterogeneous nuclear ribonucleoprotein U	255-266	824	NI/I
KAEVEGKDLPEHAVLK	1762.970	-0.001	-0.55	3	1.68E-09	5.5	0.3	126302554	Heterogeneous nuclear ribonucleoprotein U	619-634	824	NI/I
KRNFILDQTNVSAAAQR	1932.041	0.002	1.04	3	3.22E-06	4.5	0.3	126302554	Heterogeneous nuclear ribonucleoprotein U	573-589	824	NI
WGQKPWSQHYHQGYY	1964.883	0.002	1.09	2	1.17E-11	4.2	0.4	126302554	Heterogeneous nuclear ribonucleoprotein U	810-824	824	NI/I
AILLGLAVTAMKSRP	1540.924	0.000	0.07	2	2.52E-10	3.9	0.5	20270389	HIG1 domain family, member 2A	92-106	106	NI
GIAEEPEIQMV	1215.593	-0.001	-1.07	1	2.16E-04	2.0	0.4	15277507	Histone H4 transcription factor	507-517	517	NI
APWIEQEGPEYWDGETR	2062.914	-0.003	-1.42	2	2.22E-08	5.6	0.4	717123	HLA-A2	73-89	365	NI
FIAVGYVDDTQF	1374.658	-0.001	-0.47	2	1.52E-07	3.5	0.4	717123	HLA-A2	46-57	365	I
IAVGYVDDTQ	1080.521	0.003	2.40	1	3.46E-05	1.9	0.2	717123	HLA-A2	47-56	365	NI/I
IAVGYVDDTQF	1227.589	0.002	1.32	2	3.05E-05	3.0	0.4	717123	HLA-A2	47-57	365	NI/I
IAVGYVDDTQFVRF	1629.827	0.002	1.01	2	6.23E-09	3.7	0.4	717123	HLA-A2	47-60	365	I
IAVGYVDDTQFVRFD	1744.854	0.001	0.50	2	8.58E-09	4.0	0.6	717123	HLA-A2	47-61	365	NI/I
VDDTQFVRFDSD	1443.639	-0.003	-1.81	2	4.12E-08	3.8	0.1	717123	HLA-A2	52-63	365	NI/I
VETRPAGDGTFQ	1277.612	0.000	-0.04	2	3.34E-04	2.7	0.3	717123	HLA-A2	255-266	365	I
YAYDGKDY	994.415	0.000	0.28	1	8.13E-04	2.1	0.2	717123	HLA-A2 [g]	140-147	365	NI/I

HLA-B51, or -Cw1 ligand sequence	Experimental mass [a]	∆Mass [b]	∆Mass [c]	z	P (pep) [d]	Xcorr [e]	∆Cn [e]	Gi accession	Protein	Position	Length of protein	Sample [f]
SWTAADTAAQITQR	1519.750	-0.003	-1.67	2	1.81E-09	3.6	0.4	63252969	HLA-B27	131-144	181	NI
GSHSMRYFYTA	1319.584	0.001	1.07	2	4.74E-05	2.7	0.2	553533	HLA-B51	25-35	270	I
ISVGYVDDTQFVRF	1645.822	-0.002	-0.98	2	6.66E-09	3.9	0.4	386912	HLA-Cw1	47-60	366	NI
KLAPITYPQGLA	1271.736	-0.002	-1.51	2	2.21E-05	2.8	0.4	82408216	Human Rac3 In Complex With Gdp	133-144	192	NI/I
DIVLTQSPASLA	1214.663	0.000	-0.22	1	6.53E-03	2.1	0.2	418845	Ig kappa chain precursor	21-32	140	I
MVLGPAAYNVPLPK	1469.818	-0.002	-1.28	2	1.29E-04	3.7	0.4	154354964	Inner membrane protein	88-101	758	NI
ASYTWSDAAAAFAGLSREEA	2073.951	-0.001	-0.35	2	9.37E-13	5.1	0.6	23821023	Interleukin 4 induced 1	396-415	567	NI/I
FIFQQPEAPMEGFQL	1781.857	0.002	0.95	2	4.09E-08	3.4	0.3	6912478	Karyopherin alpha 6	522-536	536	NI
LPTERFSPV	1045.568	-0.002	-1.99	2	3.49E-03	2.5	0.2	38569491	KIAA0999 protein	556-564	1263	NI
SADTLWGIQKELQF	1635.838	-0.001	-0.52	2	1.43E-04	2.9	0.3	5031857	Lactate Dehydrogenase A	319-332	332	NI
LPAPPAPPVF	1005.577	0.003	2.85	1	7.66E-05	2.4	0.3	25091210	Lysine N-methyltransferase	542-531	1291	NI
GGPAPPPYSEV	1070.515	0.000	0.04	1	5.41E-04	1.8	0.3	5803056	Lysosomal multispanning membrane protein 5	252-262	262	NI
GPAPPPYSEV	1013.494	-0.001	-0.84	1	5.00E-05	1.7	0.3	5803056	Lysosomal multispanning membrane protein 5	253-262	262	NI
KTPEGGPAPPPYSEV	1525.753	-0.001	-0.51	2	1.71E-10	4.4	0.5	5803056	Lysosomal multispanning membrane protein 5	248-262	262	NI/I
LPSKTPEGGPAPPPYSEV	1822.922	-0.001	-0.47	2	8.23E-11	4.2	0.5	5803056	Lysosomal multispanning membrane protein 5	245-262	262	NI
LSLPSKTPEGGPAPPPYSEV	2023.038	0.005	2.59	2	2.94E-09	4.5	0.4	5803056	Lysosomal multispanning membrane protein 5	243-262	262	NI/I
PSKTPEGGPAPPPYSEV	1709.838	0.001	0.38	2	8.60E-10	4.8	0.5	5803056	Lysosomal multispanning membrane protein 5	246-262	262	NI/I
SKTPEGGPAPPPYSEV	1612.785	-0.001	-0.83	2	3.92E-09	4.5	0.5	5803056	Lysosomal multispanning membrane protein 5	247-262	262	NI/I
TPEGGPAPPPYSEV	1397.658	-0.003	-1.95	2	5.84E-06	3.2	0.4	5803056	Lysosomal multispanning membrane protein 5	249-262	262	NI/I
AKVAVLGASGGIGQPLSL	1637.958	0.005	3.32	2	2.16E-08	4.3	0.4	21735621	Malate dehydrogenase	25-42	338	NI/I
LLKNSPLVSRLTL	1453.910	0.002	1.51	2	1.17E-07	3.2	0.2	21735621	Malate dehydrogenase	43-55	338	I
LPAGPPPQI	889.514	-0.001	-0.60	1	8.37E-04	2.1	0.1	167736389	MAPK activating protein PM20	52-60	152	NI
MPVPTTPEF	1018.491	0.001	1.20	1	2.17E-03	1.7	0.1	132626688	Mediator of DNA damage checkpoint 1	1692-1700	2089	NI
LPSKSSSPNSPLPM	1441.736	-0.001	-0.49	2	2.17E-08	4.0	0.5	88909230	MYCPP	722-735	1863	NI
VTLGEKMTEEE	1265.593	-0.002	-1.53	2	2.62E-05	2.8	0.4	17986258	Myosin light chain 6	114-124	151	NI
AMFDQSQIQEFKEAF	1818.837	-0.002	-1.21	2	1.31E-06	3.7	0.4	15809016	Myosin regulatory light chain MRCL2	24-38	172	NI
AMFDQSQIQEFKEAFNM	2063.920	0.005	2.20	2	2.32E-09	4.0	0.5	15809016	Myosin regulatory light chain MRCL2	24-40	172	NI
DEEATGTIQEDYLREL	1881.871	0.000	0.12	2	2.92E-07	3.8	0.4	15809016	Myosin regulatory light chain MRCL2	111-126	172	NI
FDQSQIQEFKEAFNM	1861.843	0.002	1.09	2	7.62E-13	4.3	0.4	15809016	Myosin regulatory light chain MRCL2	26-40	172	NI
YREAPIDKKGNF	1437.748	0.003	1.97	2	8.42E-06	2.7	0.3	15809016	Myosin regulatory light chain MRCL2	143-154	172	NI
AGDGSDEEVDGKADGAEAKPAE	2117.911	-0.002	-1.16	3	5.78E-12	5.1	0.3	12667788	Myosin, heavy polypeptide 9, non-muscle	1939-1960	1960	NI
AKLMATLRNTNPNF	1590.842	0.002	0.99	2	4.29E-08	3.0	0.3	12667788	Myosin, heavy polypeptide 9, non-muscle	655-668	1960	NI
DEEVDGKADGAEAKPAE	1730.772	0.001	0.35	2	4.67E-07	4.8	0.3	12667788	Myosin, heavy polypeptide 9, non-muscle	1944-1960	1960	NI
DGKADGAEAKPAE	1258.591	-0.002	-1.56	2	2.04E-07	3.5	0.3	12667788	Myosin, heavy polypeptide 9, non-muscle	1948-1960	1960	NI
DGSDEEVDGKADGAEAKPAE	1989.852	0.002	0.92	3	2.32E-09	5.2	0.5	12667788	Myosin, heavy polypeptide 9, non-muscle	1941-1960	1960	NI
GKKRHEMPPHIYAITDTAYR	2384.229	-0.005	-2.12	3	2.45E-10	5.7	0.5	12667788	Myosin, heavy polypeptide 9, non-muscle	140-159	1960	NI
GSDEEVDGKADGAEAKPAE	1874.825	0.001	0.53	2	1.44E-09	4.6	0.4	12667788	Myosin, heavy polypeptide 9, non-muscle	1942-1960	1960	NI
KLVWVPSDK	1071.620	0.000	0.26	2	8.26E-04	2.9	0.2	12667788	Myosin, heavy polypeptide 9, non-muscle	30-38	1960	NI
RGDLPFVVPRR	1311.764	0.001	0.82	3	3.08E-04	3.7	0.2	12667788	Myosin, heavy polypeptide 9, non-muscle	1923-1933	1960	NI
SDEEVDGKADGAEAKPAE	1817.804	0.000	0.03	3	1.83E-08	4.5	0.3	12667788	Myosin, heavy polypeptide 9, non-muscle	1943-1960	1960	NI
SGFEPASLKEEVGEEAIVE	2019.976	-0.001	-0.51	2	2.49E-05	4.5	0.5	12667788	Myosin, heavy polypeptide 9, non-muscle	39-57	1960	NI
SGFEPASLKEEVGEEAIVEL	2133.060	-0.002	-0.96	2	1.72E-05	3.6	0.4	12667788	Myosin, heavy polypeptide 9, non-muscle	39-58	1960	NI
VDKNFINNPLAQADWAAKKL	2256.214	-0.001	-0.38	3	3.94E-11	5.5	0.4	12667788	Myosin, heavy polypeptide 9, non-muscle	12-31	1960	NI
VSHLLGINVTDFTR	1571.854	-0.002	-1.58	2	4.20E-05	2.8	0.2	12667788	Myosin, heavy polypeptide 9, non-muscle	374-387	1960	NI
VVINPYKNLPIYSEE	1777.937	-0.001	-0.40	2	3.67E-09	4.1	0.4	12667788	Myosin, heavy polypeptide 9, non-muscle	119-133	1960	NI
YVDKNFINNPLAQADWAAKKL	2419.277	-0.001	-0.49	3	4.92E-14	6.5	0.5	12667788	Myosin, heavy polypeptide 9, non-muscle	11-31	1960	NI
FILQEDAADSF	1255.584	0.000	-0.09	1	1.27E-03	2.1	0.2	23831195	Myosin-If	832-842	1098	NI

HLA-B51, or -Cw1 ligand sequence	Experimental mass [a]	ΔMass [b]	ΔMass [c]	z	P (pep) [d]	Xcorr [e]	ΔCn [e]	Gi accession	Protein	Position	Length of protein	Sample [f]
MPYAMTPIFDEK	1442.669	-0.001	-0.96	2	2.52E-06	3.6	0.4	62510570	NNP73	1614-1625	2785	NI
FPSSPLRIPGGNIYI	1630.895	0.001	0.35	2	3.77E-07	3.4	0.4	225756	Nuclear phosphoprotein	792-806	928	NI
SRILVSIGESFGTSEKFQK	2113.129	-0.004	-1.83	3	1.06E-11	6.7	0.5	225756	Nuclear phosphoprotein	829-847	928	NI
FVIQEGAEPFPVGRSSL	1832.954	0.003	1.72	2	2.00E-09	4.6	0.4	120660112	PHC2 protein	548-564	830	NI
GQLEHVQPWSV	1279.643	0.001	0.40	1	1.04E-05	2.2	0.4	11321601	Phosphofructokinase	774-784	784	NI
DPYTGQSV	866.389	0.000	0.07	1	1.91E-05	1.7	0.3	41322916	Plectin 1	3244-3251	4684	I
SLLQDGEFSM	1126.509	0.001	1.15	1	4.83E-05	2.0	0.3	4826898	Profilin 1	77-86	140	I
DDLGKDEL	904.426	0.003	3.21	1	5.98E-04	1.8	0.1	119621354	Protein disulfide isomerase	252-259	259	I
IEEHATKLSRTKEEL	1783.955	0.000	-0.18	3	1.34E-06	3.6	0.3	4758304	Protein disulfide isomerase A4	631-645	645	I
VLLKARLVPA	1079.730	0.000	-0.21	2	5.09E-05	2.7	0.2	147647364	Protein FAM171B	19-28	828	NI/I
VPLPPNLKPAPPPTI	1550.930	-0.001	-0.39	2	2.29E-08	2.8	0.3	20141631	Protein SON	2075-2089	2426	NI
IPVSQPGMAD	1014.492	-0.002	-1.60	1	1.35E-03	2.1	0.2	89191848	Protein YIF1B	25-34	314	NI
GRTFNLTAGSLESTEPIYVYK	2346.198	0.001	0.54	2	8.14E-13	4.5	0.5	190341074	Pyridoxal-dependent decarboxylase	660-680	788	NI
HLYRGIFPVL	1214.704	-0.001	-0.97	2	5.43E-04	2.6	0.3	33286418	Pyruvate kinase	464-473	531	NI
VVFDDSEPVQRIL	1516.801	0.005	3.38	2	1.37E-06	3.3	0.4	19923399	Ras-GAP SH3 binding protein	344-356	449	NI/I
FVLAPEGSVANKF	1378.737	0.002	1.62	2	2.84E-05	3.6	0.3	5031703	Ras-GTPase-activating protein	112-124	466	I
ARVITEEEKNFK	1463.785	0.003	1.88	2	2.09E-03	3.7	0.2	15431295	Ribosomal protein L13	166-177	211	NI
ALDVANKIGII	1126.683	-0.001	-0.75	2	1.56E-03	2.6	0.2	17105394	Ribosomal protein L23a	146-156	156	NI
DALDVANKIGII	1241.710	-0.001	-0.61	2	1.60E-04	2.6	0.2	17105394	Ribosomal protein L23a	145-156	156	NI/I
LDVANKIGII	1055.646	-0.002	-1.85	2	1.20E-04	3.0	0.2	17105394	Ribosomal protein L23a	147-156	156	NI/I
LKIEGVYARDETEF	1669.843	0.001	0.36	2	2.01E-08	3.5	0.2	16117791	Ribosomal protein L35a	28-41	110	NI
GRMHAPGKGLSQSALPYR	1926.013	-0.002	-1.11	3	1.17E-11	5.3	0.4	4506685	Ribosomal protein S13	2-19	151	NI
VRMNVLADALK	1229.703	-0.002	-1.26	2	6.43E-05	3.1	0.3	14165469	Ribosomal protein S15a	2-12	130	NI/I
NLQVTQPTV	999.547	-0.001	-1.28	1	3.80E-03	2.2	0.3	4506693	Ribosomal protein S17	116-124	135	NI
VVKVANVSLLALYK	1516.946	-0.003	-1.99	2	9.43E-10	3.6	0.4	4506701	Ribosomal protein S23	122-135	143	NI
MKIFVGNVDGADTTPEEL	1935.937	-0.003	-1.51	2	2.03E-08	4.7	0.4	5454064	RNA binding motif protein	1-18	669	NI
APYPQPPSV	955.488	-0.001	-0.69	1	2.42E-05	1.8	0.1	21703710	Scotin	157-165	240	NI
VPAPVPLM	823.475	-0.001	-1.47	1	6.06E-05	1.8	0.3	118572613	Serine/arginine repetitive matrix protein	2211-2218	2752	NI
RPHTPTPGIYM	1269.641	0.005	4.00	2	2.15E-04	2.6	0.5	4759098	Serine/arginine-rich splicing factor 10	198-208	288	I
VARNPPGFAF	1075.568	0.001	0.73	2	4.87E-05	2.5	0.3	4506901	Serine/arginine-rich splicing factor 3	41-50	164	I
ASPVVFTSA	878.462	-0.001	-1.09	1	3.83E-03	2.0	0.3	7662126	SIPA1L1 protein	1432-1440	1804	NI
LPRPSLAINGSNLPRSK	1820.050	-0.002	-1.37	3	8.07E-04	4.7	0.3	166977707	SLAI1 protein	521-537	568	NI
YEQVNMRIEG	1238.583	0.003	2.54	2	1.24E-05	2.6	0.4	4507129	Small nuclear ribonucleoprotein E	36-45	92	I
YIDGALSGHLGEVL	1443.748	0.004	2.52	2	4.25E-04	3.1	0.2	4507131	Small nuclear ribonucleoprotein F	50-63	86	I
YIRGVEEEEED	1367.596	0.004	2.96	2	1.84E-04	3.0	0.2	4507131	Small nuclear ribonucleoprotein F	71-81	86	I
AMPHPGPSPGPGPSPGPIL	1762.895	-0.003	-1.98	2	1.83E-05	3.7	0.5	48255900	SNF2L2	9-27	1590	NI
LPSILPEIWPKTPSA	1648.931	-0.001	-0.79	2	5.08E-05	3.6	0.3	18087845	Sororin	37-51	252	NI
LLTTTPRPVIVEPLEQ	1806.037	0.002	0.86	2	1.55E-05	3.6	0.3	4826998	Splicing factor proline/glutamine rich	437-452	707	NI
VMILTNPVAA	1028.581	0.000	0.07	1	8.69E-03	2.2	0.1	7661666	Testin	91-100	421	NI
SLGPSLATDKS	1075.563	-0.001	-0.73	2	3.71E-05	2.8	0.3	151101292	Thioredoxin-related transmembrane protein	270-280	280	I
DPYSSAEPHV	1101.485	-0.001	-0.79	1	4.98E-05	2.1	0.3	5032179	Transcription intermediary factor	456-465	835	NI
RLDDQESPVYAA	1363.649	0.000	0.29	1	5.57E-07	2.4	0.4	23397666	Transcriptional co-repressor Sin3A	4-15	1273	NI
NLFGGEPLSYTRFSL	1700.864	0.000	0.04	2	3.59E-07	3.7	0.3	189458817	Transferrin receptor	11-25	760	NI
FLLGPRLVLA	1098.703	-0.001	-1.26	2	6.65E-04	2.8	0.3	98986464	Transmembrane emp24 domain	22-31	219	NI
VDISQQYSNTQTFTGK	1816.871	-0.001	-0.45	2	6.81E-12	4.3	0.5	20162566	TTD non-photosensitive 1 protein	158-173	179	NI
FSETGAGKHVPRAVF	1602.839	-0.002	-0.97	2	1.01E-07	2.9	0.4	14389309	Tubulin alpha 6	53-67	449	NI
ILAPAGSLPKI	1079.682	0.001	0.57	2	4.53E-05	3.0	0.3	7019551	UbiA prenyltransferase domain	328-338	338	I

HLA-B51, or -Cw1 ligand sequence	Experimental mass [a]	ΔMass [b]	ΔMass [c]	z	P (pep) [d]	Xcorr [e]	ΔCn [e]	Gi accession	Protein	Position	Length of protein	Sample [f]
VPGVRLLQDSVDF	1444.779	-0.001	-0.77	2	2.45E-05	2.6	0.4	62414289	Vimentin	74-86	466	NI/I
AGGHKVGLALELEA	1364.753	-0.002	-1.69	2	1.32E-04	2.6	0.2	42476281	Voltage-dependent anion channel	281-294	294	NI
TPYALPVIGEVR	1314.742	0.000	-0.29	2	1.96E-07	3.1	0.2	13376798	WD repeat-containing protein C2orf44	247-258	721	NI/I
RLKSSVVALDVDQPGANYR	2088.120	-0.002	-0.75	3	7.75E-06	5.1	0.3	5730124	Zinc finger protein 217	919-937	1048	NI
EALEPPLEEQQI	1395.700	0.002	1.58	1	4.83E-07	1.7	0.2	41281612	Zinc finger protein 828	801-812	812	NI

[a] Mass of monoisotopic ion in amu

[b] Difference between nominal and experimentally detected monoisotopic ions in amu

[c] Difference between nominal and experimentally detected monoisotopic ions in ppm

[d] Probability of finding a match better than the observed match by chance

[e] Sequest score function

[f] N: non-infected; I: infected

[g] All common peptides between HLA class I molecules were asigned as HLA-A2
