# Supplementary material for: Diversity of Natural Self-Derived Ligands Presented by Different HLA Class I Molecules in Transporter Antigen Processing-Deficient Cells
Source: PLoS One. 2013 Mar 26;8(3):e59118. doi: 10.1371/journal.pone.0059118 (PMC3608615; doi:10.1371/journal.pone.0059118)
Supplement: Table S4 — Summary of HLA ligands clustered by protein origin. (PDF) [file pone.0059118.s011.pdf]

Supplemental Table 4. Summary of HLA ligands clustered by protein origin

Protein	Length	Ligand	Residues	mAb [a]		
Beta Actin	375	LVVDNGSGMCK	8-18		ME1	
		LVVDNGSGMCKAGFAGD	8-24			W6/32
		LVVDNGSGMCKAGFAGDD	8-25	PA2.1		
		LVVDNGSGMCKAGFAGDDAPRAVF	8-31		ME1	
		AGFAGDDAPR	19-28	PA2.1	ME1	
		AGFAGDDAPRAVF	19-31			W6/32
		AGFAGDDAPRAVFPSIVGRPR	19-39			W6/32
		MGQKDSYVGDEAQSK	47-61		ME1	
		YNELRVAPEEHPVL	91-104		ME1	W6/32
		YNELRVAPEEHPVLL	91-105			W6/32
		LRVAPEEHPVL	94-104			W6/32
		LRVAPEEHPVLLTEAPLNPK	94-113			W6/32
		VAPEEHPVLLTEAPLNPK	96-113	PA2.1		
		LTEAPLNPKANR	105-116	PA2.1		
		LTEAPLNPKANREKMTQ	105-121			W6/32
		YASGRTTGIVMDSGD	143-157			W6/32
		DFEQEMATAASSSSLEKS	222-239			W6/32
		SYELPDGQVITIGNER	239-254	PA2.1		
		DLYANTVLSGGTTMYPGIADR	292-312			W6/32
		DLYANTVLSGGTTMYPGIADRMQK	292-315		ME1	
		MQKEITALAPSTMK	313-326	PA2.1		W6/32
		YDESGPSIVHRKCF	362-375			W6/32
		DESGPSIVHRKCF	363-375		ME1	W6/32
CD74a	296	FLYQQQGRLDKLTV	70-83	PA2.1		
		FLYQQQGRLDKLTVT	70-84	PA2.1		
		MRMATPLLMQALPMG	107-121			W6/32
		ELEDPSSGLGVTKQDLGPVPM	276-296			W6/32
		LEDPSSGLGVTKQDLGPVPM	277-296			W6/32
		EDPSSGLGVTKQDLGPVPM	278-296			W6/32
		PSSGLGVTKQDLGPVPM	280-296		ME1	
Glyceraldehyde-3-phosphate dehydrogenase	335	AAFNSGKVDIVAINDPFIDL	21-40	PA2.1		
		INDPFIDLNY	33-42	PA2.1		W6/32

		PSKIKWGDAGAEY	82-94			W6/32
		VVESTGVFTTMEK	95-107			W6/32
		GLMTTVHAITATQK	173-186	PA2.1		W6/32
		ISWYDNEFGYSNRVVDL	311-327	PA2.1	ME1	
Heterogeneous nuclear ribonucleoprotein B1	353	ALSRQEMQEVQSSR	187-200		ME1	
		SGNFGGSRNMGGP	318-330	PA2.1		
		NMGGPYGGGNYGPGGSGGSGGYGGR	326-350	PA2.1		
		GPYGGGNYGPGGSGGSGGYGGRS	329-351	PA2.1		
		YGGGNYGPGGSGGSGGYGGR	331-350	PA2.1		
Heterogeneous nuclear ribonucleoprotein U	824	GAAKEAAGKSSGPTSL	178-193		ME1	
		GYFEYIEENKYSR	255-266			W6/32
		KRNFILDQTNVSAAAQR	573-589			W6/32
		KAEVEGKDLPEHAVLK	619-634			W6/32
		VEGKDLPEHAVLK	622-634		ME1	
		TLPEVAECF	640-648	PA2.1		
		PEVAECFDE	642-650	PA2.1		
		NQSQGYNQWQQGQF	796-809	PA2.1	ME1	
		NQSQGYNQWQQGQFWGQKP	796-814	PA2.1		
		WGQKPWSQHYHQGYY	810-824		ME1	W6/32
HLA-A2	365	FIAVGYVDDTQF	46-57			W6/32
		IAVGYVDDTQ	47-56	PA2.1		W6/32
		IAVGYVDDTQF	47-57	PA2.1		W6/32
		IAVGYVDDTQFVRF	47-60	PA2.1		W6/32
		IAVGYVDDTQFVRFD	47-61			W6/32
		IAVGYVDDTQFVRFDSD	47-63	PA2.1		
		VGYVDDTQF	49-57	PA2.1		
		VDDTQFVRFDSD	52-63			W6/32
		APWIEQEGPEYWDGETR	73-89	PA2.1		W6/32
		HRVDLGTLR	98-106		ME1	
		YAYDGKDY	140-147			W6/32

		YAYDGKDYIAL	140-150	PA2.1		
		ITLTWQRDGEDQTQDTEL	237-254		ME1	
		VETRPAGDGTFQ	255-266			W6/32
		LRWEPSSQPTIPIVG	296-310		ME1	
Ig kappa chain precursor	140	DIVLTQSPASL	21-31		ME1	
		DIVLTQSPASLA	21-32			W6/32
		DIVLTQSPASLAVSLGQ	21-37		ME1	
		DIVLTQSPASLAVSLGQR	21-38		ME1	
		DIVLTQSPASLAVSLGQRA	21-39		ME1	
Lysosomal multispanning membrane protein 5	262	PSYEEALSLPSKTPEGGPAPPPYSEV	237-262		ME1	
		EEALSLPSKTPEGGPAPPPYSEV	240-262		ME1	
		EALSLPSKTPEGGPAPPPYSEV	241-262		ME1	
		LSLPSKTPEGGPAPPPYSEV	243-262		ME1	W6/32
		SLPSKTPEGGPAPPPYSEV	244-262		ME1	
		LPSKTPEGGPAPPPYSEV	245-262		ME1	W6/32
		PSKTPEGGPAPPPYSEV	246-262			W6/32
		SKTPEGGPAPPPYSEV	247-262		ME1	W6/32
		KTPEGGPAPPPYSEV	248-262		ME1	W6/32
		TPEGGPAPPPYSEV	249-262		ME1	W6/32
		GGPAPPPYSEV	252-262			W6/32
		GPAPPPYSEV	253-262			W6/32
Myosin regulatory light chain MRCL2	172	ATSNVFAMFDQSQIQEFK	18-35	PA2.1		
		AMFDQSQIQEFK	24-35	PA2.1	ME1	
		AMFDQSQIQEFKEAF	24-38	PA2.1	ME1	W6/32
		AMFDQSQIQEFKEAFNM	24-40			W6/32
		FDQSQIQEFK	26-35		ME1	
		FDQSQIQEFKEAFNM	26-40			W6/32
		PEDVIRNAF	99-107		ME1	
		DEEATGTIQEDYLREL	111-126			W6/32
		YREAPIDKKGNF	143-154			W6/32
		KGNFNYIEFTR	151-161		ME1	
		TRILKHGAKDKDD	160-172		ME1	
Myosin heavy polypeptide 9	1960	YLYVDKNFINNPLAQADWAAK	9-29		ME1	
		YVDKNFINNPLAQADWAAKKL	11-31			W6/32
		VDKNFINNPLAQADWAAKKL	12-31			W6/32
		NFINNPLAQADWAAK	15-29	PA2.1	ME1	
		KLVWVPSDK	30-38	PA2.1		W6/32

KLVWVPSDKSGFEPASLK	30-47		ME1	
SGFEPASLKEEVGEEAIVE	39-57			W6/32
SGFEPASLKEEVGEEAIVEL	39-58	PA2.1		W6/32
EEVGEEAIVELVENGK	48-63	PA2.1	ME1	
KVKVNKDDIQK	64-74	PA2.1		
VKVNKDDIQK	65-74	PA2.1	ME1	
VVINPYKNLPIYSEE	119-133		ME1	W6/32
GKKRHEMPPHIYAITDTAYR	140-159			W6/32
RHEMPPHIYAITDTAYR	143-159		ME1	
VIQYLAYVASSHK	187-199		ME1	
NTDQASMPDNTAAQK	359-373		ME1	
VSHLLGINVTDFTR	374-387		W6/32	
AKLMATLRNTNPNF	655-668		W6/32	
RGDLPFVVPR	1923-1932		ME1	
RGDLPFVVPRR	1923-1933			W6/32
GAGDGSDEEVDGKADGAEAKPAE	1938-1960		ME1	
AGDGSDEEVDGKADGAEAKPAE	1939-1960		ME1	W6/32
DGSDEEVDGKADGAEAKPAE	1941-1960			W6/32
GSDEEVDGKADGAEAKPAE	1942-1960			W6/32
SDEEVDGKADGAEAKPAE	1943-1960			W6/32
DEEVDGKADGAEAKPAE	1944-1960			W6/32
EVDGKADGAEAKPAE	1946-1960		ME1	
DGKADGAEAKPAE	1948-1960			W6/32
GKADGAEAKPAE	1949-1960	PA2.1		

[a] The mAbs used were: PA2.1 (anti-HLA-A2), ME1 (anti-HLA-B27), and W6/32 (specific for a monomorphic HLA class I determinant)
