# Supplementary material for: Diversity of Natural Self-Derived Ligands Presented by Different HLA Class I Molecules in Transporter Antigen Processing-Deficient Cells
Source: PLoS One. 2013 Mar 26;8(3):e59118. doi: 10.1371/journal.pone.0059118 (PMC3608615; doi:10.1371/journal.pone.0059118)
Supplement: Table S5 — Subcellular location of proteins with TAP-independent ligands presented by HLA-A2, -B27, and -B51 or –Cw1. (PDF) [file pone.0059118.s012.pdf]

Supplementary Table 5

Subcellular location of proteins with TAP-independent ligands presented by HLA-A2, -B27, and -B51 or –Cw1

Protein	Gi accession	Subcellular location [a]	N[er] of peptides
Beta Actin	4501885	cytoskeleton	24
CD74a	68448544	ER-Golgi-endosome [b]	7
Glyceraldehyde-3-phosphate dehydrogenase	7669492	cytosol	6
HLA-A2	717123	ER-Golgi-membrane	15
Heterogeneous nuclear ribonucleoprotein A1	133254	nucleus, cytoskeleton	5
Heterogeneous nuclear ribonucleoprotein U	126302554	nucleus, cytoskeleton	10
Myosin regulatory light chain MRCL2	15809016	cytoskeleton	11
Myosin, heavy polypeptide 9, non-muscle	12667788	cytoskeleton	30
TTD non-photosensitive 1 protein	20162566	nucleus	1
Tubulin alpha 6	14389309	cytoskeleton	3
Total peptides in these 10 proteins			112

[a] Cell location based on gene ontology analysis (http://www.geneontology.org)

[b] ER, endoplasmic reticulum
