# Supplementary material for: Diversity of Natural Self-Derived Ligands Presented by Different HLA Class I Molecules in Transporter Antigen Processing-Deficient Cells
Source: PLoS One. 2013 Mar 26;8(3):e59118. doi: 10.1371/journal.pone.0059118 (PMC3608615; doi:10.1371/journal.pone.0059118)
Supplement: Table S6 — Summary of HLA ligands identified and coverage protein from individual protein. (PDF) [file pone.0059118.s013.pdf]

Supplemental Table 6. Summary of HLA ligands identified and coverage protein from individual protein

Protein	Accession	HLA-A2		HLA-B27		HLA-B51 or HLA-Cw1		Total [a]			
		Number of peptides	% protein coverage [b]	Number of peptides	% protein coverage [b]	Number of peptides	% protein coverage [b]	Number of peptides	% protein coverage [b]	Length of protein	Proteome of secretory vesicle [c]
ABHD14B protein	30047108					1	3.8			238	
ACSL1	40807491	1	1.7			1	1.7	1	1.7	698	
Actin related protein	5031601			1	4.8					372	Yes
ADAM metallopeptidase domain 10	4557251			1	1.3					748	Yes
Adenosylhomocysteinase	9951915					1	2.3			432	
AHNAK nucleoprotein	15214688					1	9.4			149	Yes
ALG10 Protein	47125451	1	1.9							473	
Alpha 2 globin	4504345	1	7.8			1	7.8	2	12.0	142	Yes
Aspartyl aminopeptidase	17367145	1	2.9							475	
ATP synthase	5453559	1	5.6			1	6.2	2	11.8	161	Yes
ATP synthase-coupling factor 6	18644883					2	20.4			108	
ATPase	17136148					1	2.8			470	Yes
ATPase family protein	187611510					1	1.1			893	Yes
BCL-6 corepressor	183396787					1	0.5			1755	
Beta 2-microglobulin	114319011					1	8.9			124	Yes
Beta Actin	4501885	6	19.0	7	24.0	14	38.7	24	47.0	375	Yes
Beta tubulin	2119276	1	3.4			1	3.7	2	7.1	437	Yes
C5orf32 protein	18645165					1	9.3			97	
Calnexin precursor	10716563			2	2.9					592	Yes
Calreticulin precursor	4757900	1	2.9	1	2.9			1	2.9	417	Yes
Carbonyl reductase II	33150790	1	8.6							244	
Catenin	146231940					1	1.5			968	
CD74a	68448544	2	5.1	1	5.7	4	12.2	7	17.3	296	
Cdc42	13786127	1	3.4							356	Yes
Centrosomal protein 170kDa	109255228					1	1.3			1584	Yes
Chaperonin	31542947	2	3.1			2	3.1	2	3.1	573	Yes
Chromosome 1 open reading frame 77	119573678			1	8.4					154	
Class I cytokine receptor	4759328	2	3.1			1	3.1	2	3.1	636	Yes
Cofilin 1	5031635					1	5.4			166	
Cold inducible RNA binding protein	4502847					1	10.5			172	
Collagen, type IV, alpha 5	156230677	1	0.5			1	0.5	1	0.5	1691	Yes
Cyclophilin A	1633054	4	10.4							164	Yes
DEAD box polypeptide 1	4826686	1	1.9			2	3.9	2	3.9	740	Yes
DEAD box polypeptide 27	119596071	1	2.1			1	2.1	1	2.1	440	Yes
DEAD box polypeptide 47	20149629					1	4.4			455	Yes
DEAH box polypeptide 15	68509926					1	1.6			795	Yes
DEAH box polypeptide 37	29029601					1	0.8			1157	Yes
Dedicator of cytokinesis 2	31377468					2	1.6			1830	Yes
Delta globin	4504351			1	7.5	2	17.0	3	19.0	147	Yes
Dermcidin	16751921					1	10.9			110	
Dihydrolipoyl transacylase	736675	1	2.9							477	
Dipeptidyl peptidase 1	189083844					1	2.8			463	
DnaJ (Hsp40) homolog	5453980			1	3.0	1	3.0	1	3.0	504	
Dodecenoyl-CoA isomerase	62530384	1	3.6							302	

EF-hand domain D2	20149675					1	4.6			240	
Elongation factor Tu	1706611					1	2.7			452	Yes
Enolase 1	4503571					1	2.3			434	Yes
ETS translocation variant 3	62512143	1	2.1							512	
Eukaryotic translation elongation factor 1 alpha 1	4503471	2	5.2			1	2.4	2	5.2	462	Yes
Eukaryotic translation elongation factor 1 beta 2	4503477	1	7.6			1	5.3	2	12.9	225	Yes
Eukaryotic translation elongation factor 2	4503483	1	1.5							858	Yes
Eukaryotic translation initiation factor 3	83281438	1	3.5							258	Yes
Ewing sarcoma	4885225			1	2.4					656	
F-actin capping protein	62898013					2	9.2			286	Yes
Farnesyl Diphosphate Synthase	61680786	1	2.7							374	
FCH domain only 1 [Homo sapiens]	29789054					1	1.0			889	
Fermitin family homolog 2	29789006	1	1.9			1	1.9	1	1.9	680	
Flightless I homolog	4503743	1	1.3							1269	
FUSE-binding protein 1	17402900					1	2.8			644	
FUSE-binding protein 3	100816392					1	2.6			572	
Fusion	4826734			1	3.0					526	
Gene product	348239	1	2.5	1	3.4			2	5.9	471	
G-interferon-inducible lysosomal thiol reductase	12643406	1	4.6			1	4.6	1	4.6	261	
Glioma tumor suppressor	93141272	1	3.6			1	2.9	2	3.6	478	
Glutathione transferase	4504183	2	8.1							210	
Glyceraldehyde-3-phosphate dehydrogenase	7669492	4	16.0	1	5.1	4	14.9	6	23.3	335	Yes
GM2 ganglioside activator	39995109	2	9.8							193	
Granulysin isoform NKG5	7108344	1	7.6							145	
GRB10 interacting GYF protein	42476299					1	0.7			1299	
Heat shock 70kDa protein	5729877	1	1.5							646	Yes
Helios	119220596					1	1.7			526	Yes
Hematopoietic lineage cell-specific protein	123557	1	3.3							486	Yes
Heterogeneous nuclear ribonucleoprotein A1	133254	1	4.8			4	11.3	4	11.3	372	Yes
Heterogeneous nuclear ribonucleoprotein A3	34740329	1	5.8							378	Yes
Heterogeneous nuclear ribonucleoprotein B1	14043072	4	7.4	1	4.0			5	11.4	353	Yes
Heterogeneous nuclear ribonucleoprotein R	5031755					2	4.6			633	Yes
Heterogeneous nuclear ribonucleoprotein U	126302554	4	3.6	3	5.2	4	7.4	10	12.3	824	Yes
Heterogeneous ribonucleoprotein A1	133254			1	3.8					372	Yes
HIG1 domain family, member 2A	20270389					1	14.2			106	
Histone cluster 2	28195394	1	17.7							130	Yes
Histone H4 transcription factor	15277507	1	2.1			1	2.1	1	2.1	517	
HLA-A2	229994	7	16.7	3	14.4	9	20.4	15	44.8	270	Yes
HLA-B27	63252969			1	7.7	1	7.7	2	15.4	181	Yes
HLA-B51	553533					1	4.1			270	Yes
HLA-Cw1	386912					1	3.8			366	Yes
Human Rac3 In Complex With Gdp	82408216					1	6.3			192	Yes
Ig kappa chain precursor	418845			4	13.6	1	8.6	5	13.6	140	
Inner membrane protein	154354964					1	1.8			758	
Interferon gamma receptor 1	4557880	2	3.1							489	
Interleukin 2 receptor	4504665	1	2.5							551	Yes
Interleukin 4 induced 1	23821023			1	3.3	1	3.5	2	3.5	567	Yes
Interleukin enhancer-binding factor 3	24234750			1	1.2					894	Yes
Interleukin-1 receptor-associated kinase 1	68800243	1	2.0							712	

Karyopherin alpha 6	6912478	1	2.8			1	2.8	1	2.8	536	Yes
KIAA0999 protein	38569491					1	0.7			1263	
Lactate Dehydrogenase A	5031857	1	4.2			1	4.2	1	4.2	332	
Lactate Dehydrogenase B	4557032	1	3.6							334	
Lectin, mannose-binding, 1 precursor	5031873			1	2.5					510	
Leukocyte membrane antigen	56550061	1	3.0							299	
Low density lipoprotein receptor	4505021	1	2.5							357	Yes
Lysine N-methyltransferase	25091210					1	0.8			1291	
Lysosomal multispanning membrane protein 5	5803056			8	9.9	8	7.6	12	9.9	262	
Lysosomal-associated membrane protein 2	4504957			1	2.9					410	
Malate dehydrogenase	21735621					2	9.2			338	Yes
MAPK activating protein PM20	167736389					1	5.9			152	
Mediator of DNA damage checkpoint 1	132626688					1	0.4			2089	
Methylenetetrahydrofolate dehydrogenase	115206	1	1.1							935	
MYCPP	88909230					1	0.8			1863	
Myosin light chain 6	17986258					1	7.3			151	Yes
Myosin regulatory light chain 2	284326	1	5.9							204	Yes
Myosin regulatory light chain MRCL2	15809016	2	10.5	6	22.1	5	26.2	11	45.3	172	Yes
Myosin, heavy polypeptide 9, non-muscle	12667788	5	2.0	10	6.0	16	7.3	30	10.5	1960	Yes
Myosin-If	23831195					1	1.0			1098	Yes
NNP73	62510570					1	0.4			2785	
Nuclear phosphoprotein	225756					2	3.7			928	
Nucleoporin	3135319	1	3.5							318	
Nucleoporin 210	27477134	1	0.5							1887	
Nucleoside-diphosphate kinase 4	4826862			1	8.0					187	Yes
Paraspeckle protein 1	109240550			2	3.3					523	
PHC2 protein	120660112					1	2.0			830	
Phosphofructokinase	11321601					1	1.4			784	
Phosphoglycerate kinase 1	4505763	1	2.6							417	Yes
Plectin 1	41322916					1	0.2			4684	Yes
Poliovirus receptor-related 1	42560237	1	4.3							517	
Profilin 1	4826898	1	7.1			1	7.1	1	7.1	140	Yes
Prostatic binding protein	4505621	1	7.0							187	
Protein disulfide isomerase	119621354			1	6.2	1	3.1	2	6.2	259	Yes
Protein disulfide isomerase A4	4758304					1	2.3			645	Yes
Protein FAM171B	147647364					1	1.2			828	
Protein SON	20141631					1	0.6			2426	
Protein YIF1B	89191848					1	3.2			314	
Pyridoxal-dependent decarboxylase	190341074					1	2.7			788	
Pyruvate kinase	33286418					1	1.9			531	Yes
Ras-GAP SH3 binding protein	19923399					1	2.9			449	
Ras-GTPase-activating protein	5031703					1	2.8			466	
Ribosomal protein L13	15431295					1	5.7			211	Yes
Ribosomal protein L23a	17105394					3	7.1			156	Yes
Ribosomal protein L35a	16117791					1	12.7			110	Yes
Ribosomal protein L7	15431301			1	5.2					248	Yes
Ribosomal protein L9	15431303			1	6.3					192	Yes
Ribosomal protein S11	4506681			1	9.5					158	Yes
Ribosomal protein S13	4506685					1	11.9			151	Yes

Ribosomal protein S15a	14165469			1	8.5	1	8.5	1	8.5	130	Yes
Ribosomal protein S17	4506693	1	6.7			1	6.7			135	Yes
Ribosomal protein S2	15055539	1	4.4							293	Yes
Ribosomal protein S23	4506701					1	9.8			143	Yes
Ribosomal protein S3a	4506723	1	6.4							264	Yes
Ribosomal protein S8	4506743	2	5.8							208	Yes
RNA binding motif protein 14	5454064			1	1.8	1	2.7	2	4.5	669	
Scotin	21703710					1	3.8			240	
Serine/arginine repetitive matrix protein	118572613					1	0.3			2752	
Serine/arginine-rich splicing factor 10	4759098					1	3.8			288	Yes
Serine/arginine-rich splicing factor 3	4506901					1	6.1			164	Yes
Signal recognition particle 14kDa	149999611	1	11.0							136	
SIPA1L1 protein	7662126					1	0.5			1804	
SLAI1 protein	166977707					1	3.0			568	
Small nuclear ribonucleoprotein E	4507129					1	10.9			92	Yes
Small nuclear ribonucleoprotein F	4507131					2	29.1			86	Yes
SNF2L2	48255900					1	1.2			1590	
Sororin	18087845					1	6.0			252	
Splicing factor 1	119594698			1	1.9					587	Yes
Splicing factor proline/glutamine rich	4826998			1	1.3	1	2.3	2	3.6	707	Yes
Synaptotagmin binding	119569012			1	2.9					588	
TBP-associated factor 15	21327701	1	2.7							592	
Testin	7661666					1	2.4			421	
Thioredoxin-related transmembrane protein	151101292					1	3.9			280	
TMEM9 domain family	11034855	1	4.5							198	
Transcription intermediary factor	5032179					1	1.2			835	
Transcriptional co-repressor Sin3A	23397666					1	0.9			1273	Yes
Transferrin receptor	189458817	1	1.4			1	2.0	2	2.0	760	Yes
Transmembrane 9 protein	164519076			1	2.3					642	
Transmembrane emp24 domain	98986464					1	4.6			219	
Transmembrane protein 41B	66392190	1	3.1							291	Yes
TTD non-photosensitive 1 protein	20162566	1	8.9	1	8.9	1	8.9	1	8.9	179	
Tubulin alpha 6	14389309	2	8.0	2	8.0	1	3.3	3	9.6	449	Yes
UbiA prenyltransferase domain	7019551	1	3.3			1	3.3	1	3.3	338	
Vimentin	62414289			1	2.8	1	2.8	2	5.6	466	Yes
Voltage-dependent anion channel	42476281					1	4.8			294	
WD repeat domain 41	42716287	1	3.5							459	Yes
WD repeat-containing protein C2orf44	13376798					1	1.7			721	Yes
Zinc finger protein 217	5730124					1	1.8			1048	Yes
Zinc finger protein 828	41281612					1	1.5			812	Yes

[a] Total of different peptides and % protein coverage detected both HLA-A2, -B27 and B51 or -Cw1 cell extracts

[b] Only the longer peptide was included when nested peptides were detected

[c] From human neutrophis (32)
