# Supplementary material for: Diversity of Natural Self-Derived Ligands Presented by Different HLA Class I Molecules in Transporter Antigen Processing-Deficient Cells
Source: PLoS One. 2013 Mar 26;8(3):e59118. doi: 10.1371/journal.pone.0059118 (PMC3608615; doi:10.1371/journal.pone.0059118)
Supplement: Table S7 — Summary of predominant cleavaged residues by peptidases in TAP-independent ligands. (PDF) [file pone.0059118.s014.pdf]

Supplemental Table 7

Summary of predominant cleavaged residues by peptidases in TAP-independent ligands

Cleavaged residue	HLA-A2		HLA-B27		HLA-B51, -Cw1		Total HLA alleles	
	P_1 N-end [a]	P_1 C-end [a]	P_1 N-end [a]	P_1 C-end [a]	P_1 N-end [a]	P_1 C-end [a]	P_1 N-end [a]	P_1 C-end [a]
K/R	33 [b]	23	34	42	27	24	33	30
L/F	24	29	20	30	24	27	24	28
Σ	57	52	54	72	51	51	57	58

[a] from figures 6 and 7.

[b] Data are expressed as percentage of total cleavages.
